# Tumor‐Intrinsic ARHGEF3 Enhances Antitumor Immunity by Promoting T‐Cell Infiltration and Limiting Myeloid Cell‐Mediated Immunosuppression

**DOI:** 10.1002/advs.202523895

**Published:** 2026-04-23

**Authors:** Yue Li, Lan Wang, Zihao Zhang, Chunmei Qian, Ning Li, Wei Huang, Qian Ba, Xiaojian Liu, Mayu Sun

**Affiliations:** ^1^ Science and Technology Innovation Center Shanghai Municipal Hospital of Traditional Chinese Medicine Shanghai University of Traditional Chinese Medicine Shanghai China; ^2^ The Third Affiliated Hospital of Beijing University of Chinese Medicine Beijing China; ^3^ CAS Key Laboratory of Nutrition Metabolism and Food Safety Shanghai Institute of Nutrition and Health University of Chinese Academy of Sciences Chinese Academy of Sciences Shanghai China; ^4^ Department of Pharmacy The First Affiliated Hospital of USTC Division of Life Sciences and Medicine University of Science and Technology of China Hefei Anhui China; ^5^ Institute of Image Processing and Pattern Recognition Key Laboratory of System Control and Information Processing Ministry of Education of China Shanghai Jiao Tong University Shanghai China

**Keywords:** ARHGEF3, fatty acid metabolism, myeloid cell‐mediated immunosuppression, T‐cell infiltration, tumor microenvironment

## Abstract

A lack of effective antitumor T‐cell immunity often drives immune evasion and immunotherapy resistance. Here, we demonstrated that tumor‐intrinsic ARHGEF3 reprogrammed the tumor microenvironment into a T‐cell‐inflamed state, resulting in potent antitumor effects. Mechanistically, ARHGEF3 functioned as a guanine nucleotide exchange factor that activated the RHOA‐ROCK‐PTEN cascade and inhibited AKT signaling. This inhibition upregulated IRF1‐dependent chemokines CXCL10 and CXCL11 to drive T‐cell infiltration, while suppressing FASN‐mediated fatty acid synthesis to limit myeloid cell‐mediated immunosuppression. The dual effects elicited robust T‐cell immunity and overcame tumor resistance to immunotherapy. In human tumors, *ARHGEF3* expression correlated positively with T‐cell‐inflamed signatures, improved clinical outcomes, and responsiveness to immunotherapy. Collectively, these findings identify ARHGEF3 as a key modulator linking chemokine signaling with lipid availability to shape T‐cell immunity, offering a promising therapeutic strategy to overcome immunotherapy resistance.

## Graphical abstract

ARHGEF3 is broadly downregulated across human cancers and correlates with patient prognosis. Tumor‐intrinsic ARHGEF3 activates the RHOA–ROCK–PTEN cascade to inhibit AKT signaling, thereby promoting chemokine‐driven T‐cell infiltration and relieving lipid‐mediated myeloid immunosuppression. These coordinated effects remodel a T cell‐inflamed microenvironment, support durable antitumor immunity, and overcome resistance to immunotherapy.

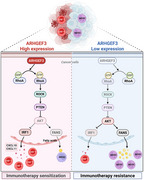

**Abbreviations**
MDSCs: myeloid‐derived suppressor cells
ICB: immune checkpoint blockade
TME: tumor microenvironment
sgRNA: single‐guide RNA
RT‐qPCR: quantitative real‐time PCR
TCM: tumor cells‐derived conditioned medium
BLCA: bladder urothelial carcinoma
KICH: kidney chromophobe
SKCM: skin cutaneous melanoma
DLBC: diffuse large B‐cell lymphoma
LAML: acute myeloid leukemia
MESO: mesothelioma
SARC: sarcoma
THYM: thymoma
UVM: uveal melanoma
DCs: dendritic cells
NKs: natural killer cells
FFA: free fatty acid
GEP: gene‐expression profile
TLS: tertiary lymphoid structures.

## 1 Introduction

Despite the transformative impact of cancer immunotherapy, most patients derive limited benefit owing to primary or acquired resistance [1]. Non‐responders are frequently characterized by “immune‐cold” tumors with scant T‐cell infiltration and dominant myeloid cell‐mediated immunosuppression [2]. Within such microenvironments, immunosuppressive myeloid populations, including tumor‐associated macrophages and myeloid‐derived suppressor cells (MDSCs), enforce immune evasion and blunt cytotoxic T‐cell function [2, 3]. Targeting these lineages has therefore emerged as a principal strategy to convert cold tumors into T‐cell–inflamed states that respond to immune checkpoint blockade (ICB) [4].

In the tumor microenvironment (TME), various chemokines that are secreted by tumor cells or tumor‐infiltrating immune cells have been reported to contribute to the trafficking of immune cells, including both effector T cells and suppressive populations [5]. Beyond chemokine‐driven migration, local proliferation and differentiation also expand immune‐cell numbers. Cancer cells preferentially consume glucose to sustain aerobic glycolysis and channel carbon into de novo lipogenesis, which establishes a glucose‐poor, lipid‐rich milieu that undermines T‐cell survival and effector function [6, 7, 8]. However, this environment is not uniformly suppressive for all immune cells. Myeloid populations such as MDSCs, increase fatty‐acid uptake and activate fatty‐acid oxidation, thereby promoting their expansion and enforcing an immunosuppressive phenotype [9]. Such fatty‐acid–rich conditions have been linked to resistance to cancer immunotherapy by sustaining MDSC activity and preventing the reinvigoration of effector T cells [10]. Accordingly, strategies that modulate chemokine axes while restraining lipid‐driven myeloid cell–mediated immunosuppression may restore T‐cell fitness and convert immune‐cold tumors into a hot state.

Rho guanine nucleotide exchange factor 3 (ARHGEF3; also known as XPLN) is a RhoA/B‐specific exchange factor that governs Rho GTPase activation. In humans, ARHGEF3 encodes a 526‐amino‐acid protein bearing an N‐terminal Dbl‐homology (DH) domain and a C‐terminal pleckstrin‐homology (PH) domain, with broad expression in brain, heart, kidney, platelets and skeletal muscle. ARHGEF3 has two established activities [11, 12]. First, as a RhoGEF, it selectively activates RhoA and RhoB but not RhoC. Second, independent of GEF function, it serves as an endogenous inhibitor of mTORC2 through cooperative engagement of its N‐terminal region and PH domain, thereby attenuating mTORC2 kinase activity and downstream AKT signaling. Through its GEF activity, ARHGEF3 activates RhoA to engage Rho‐associated coiled‐coil–containing kinases (ROCKs) [13]. This RhoA‐ROCK axis promotes activation of the lipid phosphatase PTEN, which converts PIP3 to PIP2 and thereby restrains the PI3K‐AKT‐mTOR cascade [14]. ARHGEF3 can thus limit AKT signaling both by direct inhibition of mTORC2 and indirectly via a RhoA‐ROCK‐PTEN axis. Although ARHGEF3 has been investigated in muscle regeneration and cytoskeletal control [15], its roles in cancer, particularly in shaping the immune microenvironment, remain largely undefined.

Herein, by integrating pan‐cancer analyses from The Cancer Genome Atlas with mechanistic studies in murine tumor models, we delineate the immunological roles of ARHGEF3. We show that tumor‐intrinsic ARHGEF3 reprograms the microenvironment into a T cell‐inflamed state by promoting chemokine‐driven T‐cell infiltration and by limiting fatty‐acid‐driven myeloid cell‐mediated immunosuppression, thereby restoring T‐cell function. Consequently, ARHGEF3 induces durable antitumor effects and overcomes resistance to anti‐PD‐1 therapy. Cross‐cohort analyses of human datasets link high ARHGEF3 expression to T‐cell‐inflamed transcriptional programs and improved outcomes on ICB, highlighting ARHGEF3 as an immune‐activating determinant and candidate biomarker. Our findings show that tumor‐intrinsic ARHGEF3 integrates chemokine signaling and lipid metabolism to shape antitumor immunity, providing actionable avenues for therapeutic intervention.

## 2 Results

### 2.1 *ARHGEF3* Expression is Downregulated in Multiple Cancer Types and Correlates with Patient Prognosis

**FIGURE 1:**
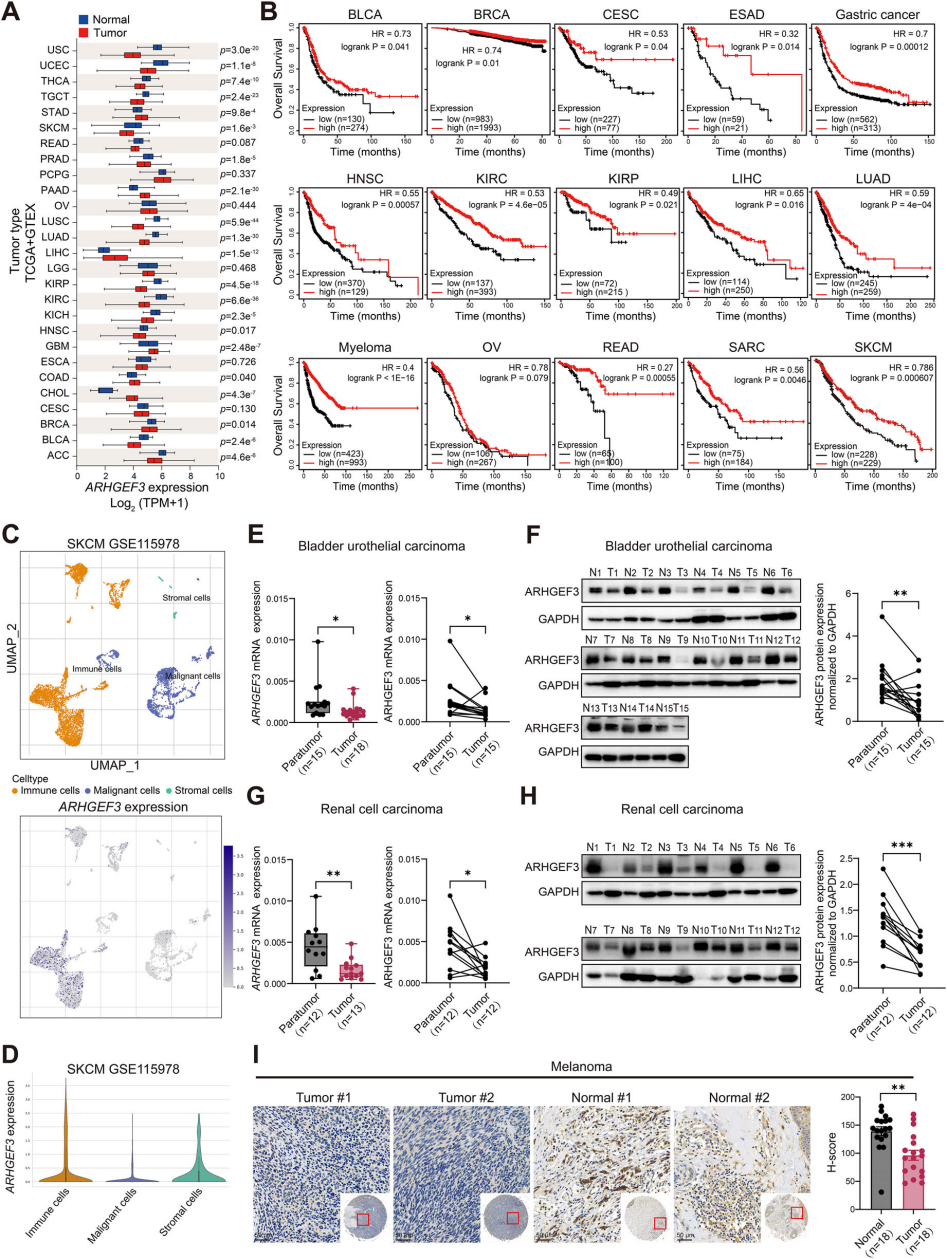
*ARHGEF3* is downregulated in tumors, and higher expression predicts improved patient survival. (A) Analysis of *ARHGEF3* mRNA expression between tumors and adjacent normal tissues across TCGA‐GTEx pan‐cancer datasets. *P* values were calculated by an unequal variance *t*‐test. (B) Association of *ARHGEF3* mRNA expression with overall survival in multiple cancer cohorts. *P* values were calculated by the log‐rank (Mantel–Cox) test. (C) UMAP plot of the SKCM single‐cell dataset (GSE115978) showing cell‐type clustering (Top) and *ARHGEF3* expression (Bottom). Cell counts: immune cells (*n* = 4856), malignant cells (*n* = 2142), stromal cells (*n* = 188). (D) Violin plot showing *ARHGEF3* expression levels across annotated cell types. (E) RT‐qPCR analysis of *ARHGEF3* expression in bladder urothelial carcinoma and adjacent normal tissues; left, all samples; right, paired samples. *P* values were calculated by unpaired (left) or paired (right) Student's *t*‐test. (F) Western blot analysis of ARHGEF3 expression in paired bladder urothelial carcinoma and adjacent normal tissues; the right panel shows protein quantification. *P* values were calculated by a paired Student's *t*‐test. (G) RT‐qPCR analysis of *ARHGEF3* expression in renal cell carcinoma and adjacent normal tissues; left, all samples; right, paired samples. *p* values were calculated by unpaired (left) or paired (right) Student's *t*‐test. (H) Western blot analysis of ARHGEF3 expression in paired renal cell carcinoma and adjacent normal tissues; the right panel shows protein quantification. *P* values were calculated by a paired Student's *t*‐test. (I) Representative immunohistochemical images of ARHGEF3 in melanoma and normal skin tissues; the right panel shows protein quantification. *P* values were calculated by an unpaired Student's *t*‐test (*P < 0.05, **P < 0.01, ***P < 0.001). BLCA: Bladder urothelial carcinoma; BRCA: Breast invasive carcinoma; CESC: Cervical squamous cell carcinoma and endocervical adenocarcinoma; ESAD: Esophageal adenocarcinoma; HNSC: Head and neck squamous cell carcinoma; KIRC: Kidney renal clear cell carcinoma; KIRP: Kidney renal papillary cell carcinoma; LIHC: Liver hepatocellular carcinoma; LUAD: Lung adenocarcinoma; OV: Ovarian serous cystadenocarcinoma; READ: Rectum adenocarcinoma; SARC: Sarcoma; SKCM: Skin cutaneous melanoma.

To clarify the clinical relevance of ARHGEF3, we first examined its basal expression in normal human tissues using the GTEx database [16]. *ARHGEF3* is broadly expressed, with comparatively low levels in the liver (Figure S1). We then profiled its mRNA abundance across TCGA pan‐cancer cohorts, integrating GTEx normal tissues. *ARHGEF3* expression was significantly reduced in 15 tumor types (Figure 1A), including bladder urothelial carcinoma (BLCA), kidney chromophobe (KICH), and skin cutaneous melanoma (SKCM). Six additional cancer types, including diffuse large B‐cell lymphoma (DLBC), acute myeloid leukemia (LAML), mesothelioma (MESO), sarcoma (SARC), thymoma (THYM), and uveal melanoma (UVM), lacked matched normal tissues and were therefore not included in direct comparisons between tumors and normal samples. Across most cohorts, lower *ARHGEF3* expression was associated with markedly poorer overall survival (Figure 1B), underscoring its potential prognostic value.

To identify which intratumoral cell populations exhibit low *ARHGEF3* expression, we analyzed single‐cell RNA‐seq datasets using the Tumor Immune Single‐cell Hub (TISCH) [17]. In multiple tumor types, *ARHGEF3* was predominant in immune and stromal compartments but markedly reduced in malignant cells (Figure 1C,D, Figure S1B–K), indicating selective downregulation of *ARHGEF3* in tumor cells.

To confirm these cohort‐level observations in clinical specimens, we further examined ARHGEF3 expression in human bladder and kidney tumors. At both the mRNA and protein levels, ARHGEF3 expression was significantly decreased in tumor tissues relative to patient‐matched adjacent peritumoral tissues (Figure 1E–H). A similar reduction was also confirmed at the protein level in human lung adenocarcinoma samples (Figure S2). In melanoma tissue microarrays, immunohistochemistry likewise revealed lower ARHGEF3 protein in tumor regions compared to normal skin tissues (Figure 1I). These data support tumor cell‐intrinsic downregulation of ARHGEF3 with adverse prognostic implications.

### 2.2 Tumor‐Intrinsic ARHGEF3 Promotes Durable Antitumor Immunity and Reduces Postoperative Relapse

**FIGURE 2:**
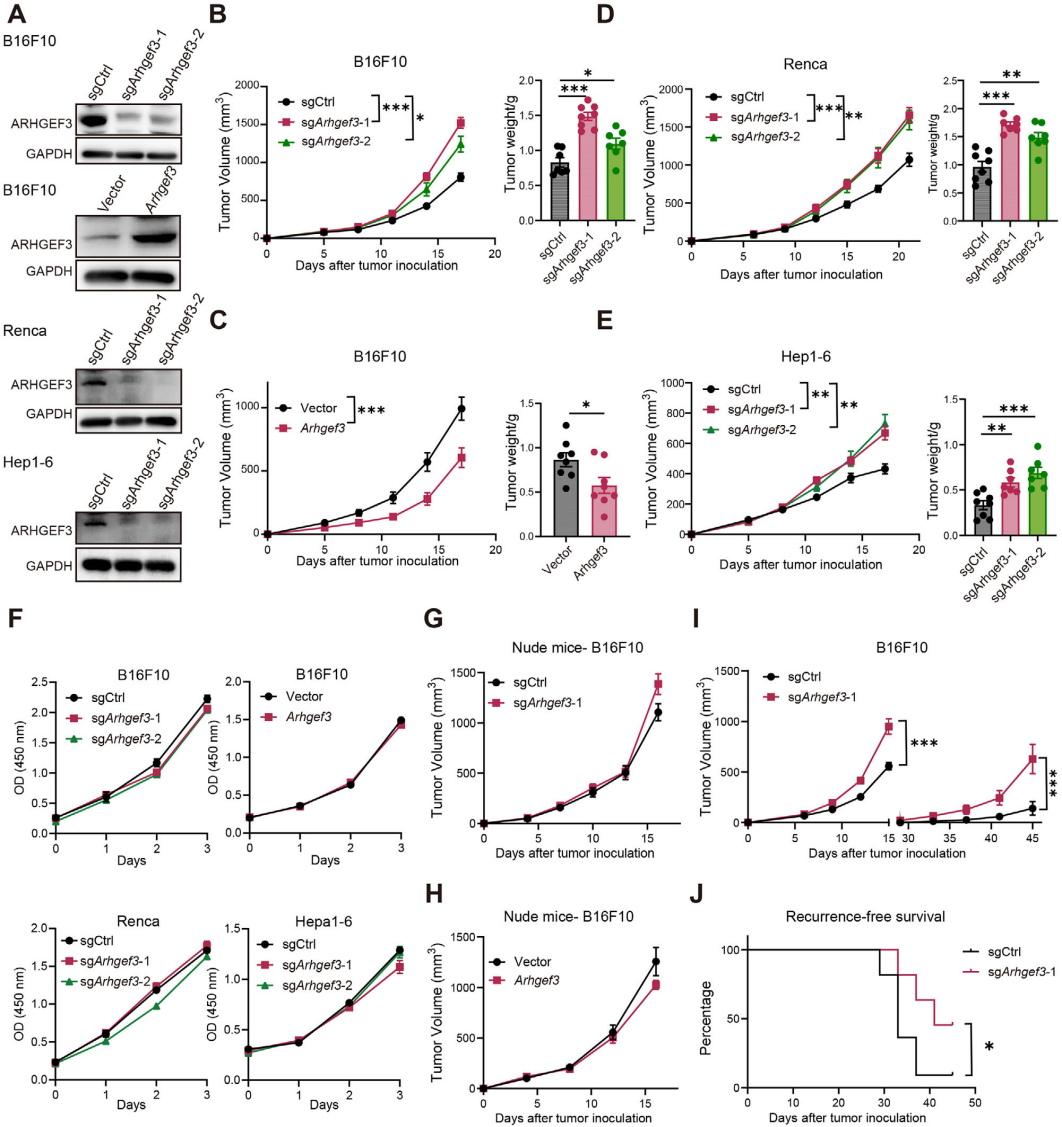
ARHGEF3 inhibits tumor growth and prevents relapse. (A) The knockout or overexpression efficiency of *Arhgef3* in B16F10, Hepa1‐6, and Renca cells was determined by western blot. (B‐E) Tumor growth curves and endpoint tumor weights for *Arhgef3*‐sgRNA B16F10 (B), *Arhgef3*‐overexpressing B16F10 (C), *Arhgef3*‐sgRNA Hepa1‐6 (D), and *Arhgef3*‐sgRNA Renca (E) tumors in immunocompetent mice. *n* = 7‐8 mice per group. (F) CCK‐8 proliferation assays showing growth curves after *Arhgef3* knockout or overexpression in the indicated cells. *n* = 3 per group. (G,H) Growth and endpoint tumor weights of *Arhgef3*‐sgRNA B16F10 (G) and *Arhgef3*‐overexpressing B16F10 (H) tumors in nude mice. n=6‐8 mice per group. (I,J) Growth of recurrent tumors (I) and recurrence‐free survival (J) in mice bearing *Arhgef3*‐overexpressing B16F10 tumors. n=11 mice per group. *P* values were calculated by unpaired Student's *t*‐test for tumor weight, two‐way ANOVA for tumor growth curves, and the log‐rank (Mantel–Cox) test for recurrence‐free survival curves (*P < 0.05, **P < 0.01, ***P < 0.001).

To define the antitumor role of ARHGEF3 in vivo, we knocked out the *Arhgef3* gene in murine Hepa1‐6 (hepatocellular carcinoma), Renca (renal cell carcinoma), and B16F10 (melanoma) cell lines using CRISPR‐Cas9 technology, and established B16F10 cells stably overexpressing *Arhgef3* (Figure 2A). These cells were implanted subcutaneously to establish tumor models. In immunocompetent mice, loss of *Arhgef3* accelerated tumor growth, whereas overexpression impeded tumor progression (Figure 2B–E). This effect was not attributable to intrinsic proliferative changes, as CCK‐8 assays showed no alteration in vitro upon *Arhgef3* knockout or overexpression (Figure 2F). Consistently, in nude mice lacking functional T cells, neither overexpression nor knockout altered tumor growth (Figure 2G,H), indicating that ARHGEF3 controls tumor growth via the intact immune system, likely through T cell‐mediated mechanisms.

We then asked whether tumor‐intrinsic ARHGEF3 can exert broader antitumor effects in vivo. In a spontaneous‐relapse B16F10 tumor model, primary lesions were resected on day 15 post‐implantation, and recurrence was longitudinally monitored. *Arhgef3* overexpression significantly reduced postoperative recurrence and slowed the expansion of recurrent tumors (Figure 2I,J), suggesting that ARHGEF3 can support antitumor immune memory. Moreover, in a B16F10 lung metastasis model, *Arhgef3* overexpression significantly reduced lung tumor burden (Figure S3). Taken together, these results identify ARHGEF3 as an immune‐linked tumor suppressor.

### 2.3 ARHGEF3 Reshapes a T Cell‐Inflamed TME

**FIGURE 3:**
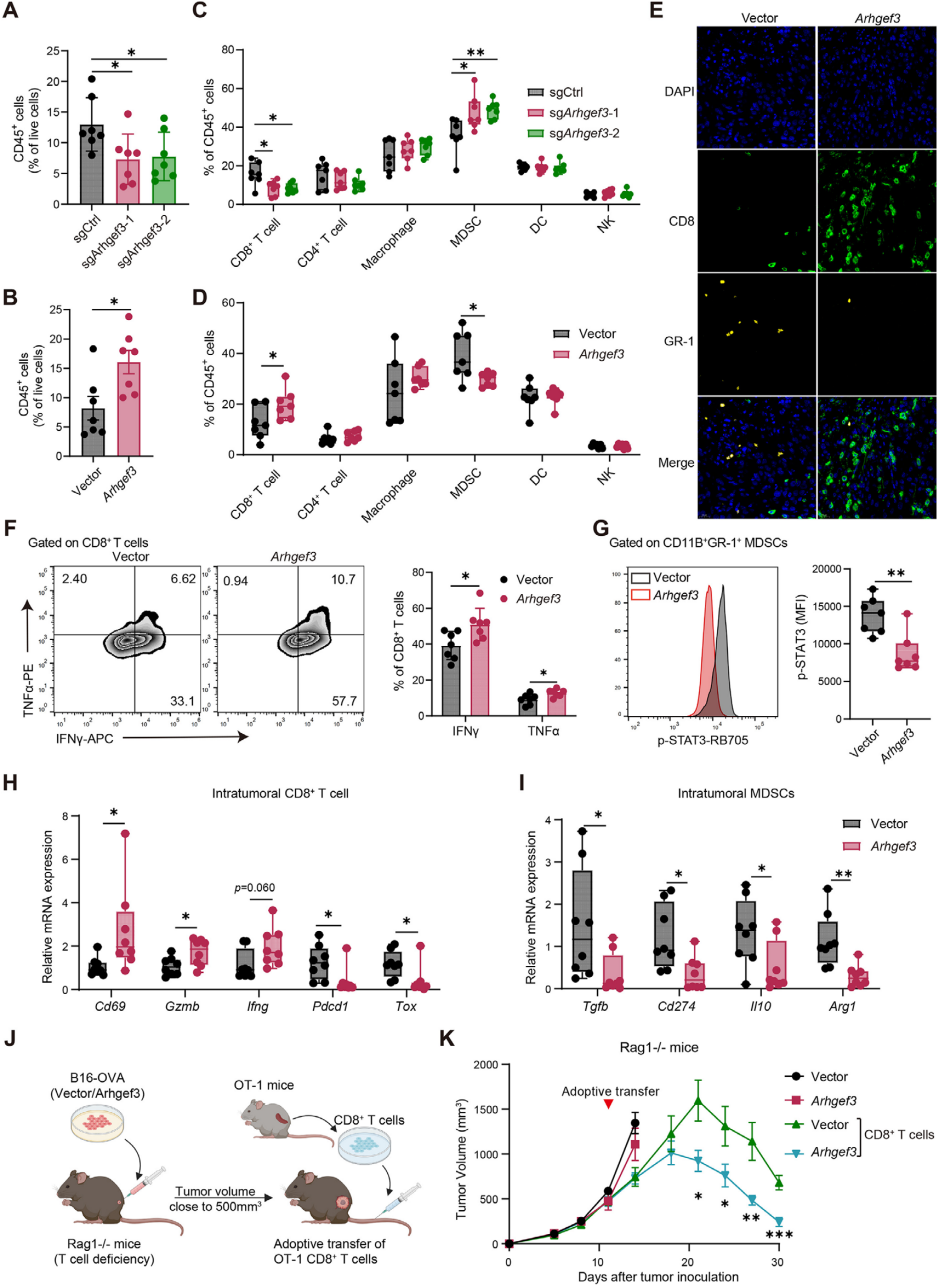
ARHGEF3 promotes a T cell–inflamed TME with reduced MDSC‐mediated suppression. (A,B) Flow cytometry analysis quantifying the proportion of infiltrating CD45^+^ cells in the TME of *Arhgef3*‐sgRNA (A) and *Arhgef3*‐overexpressing (B) B16F10 tumor‐bearing mice. *n* = 7 mice per group. (C, D) Flow cytometry analysis quantifying the proportion of infiltrating CD8^+^ T cells, CD4^+^ T cells, MDSCs, macrophages, DCs, and NKs in the TME of *Arhgef3*‐sgRNA (C) and *Arhgef3*‐overexpressing (D) B16F10 tumor‐bearing mice. *n* = 7 mice per group. (E) Representative immunofluorescence images showing the expression of CD8α, and GR‐1 in *Arhgef3*‐overexpressing B16F10 tumor tissues. (F) Representative flow cytometry plots (left) and quantification (right) showing the levels of IFN‐γ and TNF‐α in intratumoral CD8^+^ T cells. (G) Representative flow cytometry plots(left) and mean fluorescence intensity (MFI) quantification (right) showing the levels of phospho‐STAT3 in intratumoral MDSCs. (H, I) RT‐qPCR analysis of the indicated genes in intratumoral CD8^+^ T cells (H) and MDSCs (I) isolated from *Arhgef3*‐overexpressing B16F10 tumor tissues. *n* = 8 mice per group. (J) Scheme of adoptive transferring OT‐I cells in T‐cell‐deficient Rag1^−/−^ mice. (K) Growth of *Arhgef3*‐overexpressing B16F10 tumors in Rag1^−/−^ mice after indicated treatments. n=8 mice per group. *P* values were calculated by unpaired Student's *t*‐test or two‐way ANOVA (*P < 0.05, **P < 0.01, ***P < 0.001).

To determine whether ARHGEF3 remodels the antitumor immune response, we first performed GSEA on the TCGA‐SKCM cohort stratified by *ARHGEF3* expression. Among the top 10 GO terms, *ARHGEF3*‐high tumors showed enrichment for adaptive immune response, lymphocyte‐mediated immunity, leukocyte‐mediated immunity, and regulation of lymphocyte activation (Figure S4). We further analyzed the immune composition within B16F10 tumor tissues by flow cytometry. Deletion of *Arhgef3* markedly reduced the overall proportion of infiltrating CD45^+^ immune cells (Figure 3A), whereas *Arhgef3* overexpression substantially increased their accumulation (Figure 3B). Subset analysis showed that *Arhgef3* loss specifically impaired the infiltration of CD8^+^ T cells, while concomitantly driving an expansion of immunosuppressive MDSCs (Figure 3C). In contrast, *Arhgef3* overexpression drove robust CD8^+^ T‐cell infiltration and a pronounced reduction in MDSCs (Figure 3D). Immunofluorescence staining corroborated these findings, showing a significant increase in intratumoral CD8^+^ T cells (CD8α) and a marked decrease in MDSCs (GR‐1) in *Arhgef3*‐overexpressing B16F10 and Hepa1‐6 tumors (Figure 3E, Figure S5). Other immune cells, including CD4^+^ T cells, macrophages, dendritic cells (DCs), and natural killer cells (NKs), showed no consistent differences upon ARHGEF3 modulation (Figure 3C,D).

We next assessed the functional status of tumor‐infiltrating CD8^+^ T cells and MDSCs. In *Arhgef3*‐overexpressing B16F10 tumors, CD8^+^ T cells produced higher levels of IFN‐γ and TNF‐α (Figure 3F), whereas MDSCs exhibited lower levels of phospho‐STAT3 (p‐STAT3) (Figure 3G), a hallmark of an immunosuppressive state. To further define functional states, we sorted CD8^+^ T cells and MDSCs from B16F10 tumor tissues and performed RT‐qPCR. Compared with the control group, CD8^+^ T cells in *Arhgef3*‐overexpressing tumors showed increased expression of activation and effector markers (*Cd69*, *Gzmb* and *Ifng*) and decreased expression of exhaustion markers (*Pdcd1* and *Tox*) (Figure 3H). Conversely, tumor‐infiltrating MDSCs displayed reduced levels of immunosuppressive mediators (*Tgfb*, *Cd274*, *Il10*, and *Arg1*) (Figure 3I).

Given that MDSCs restrain cytotoxic T‐cell activity, we next tested whether the antitumor effect of ARHGEF3 depends on CD8^+^ T cells. We used recombinase‐activating gene 1‐deficient (Rag1^−/−^) mice, which lack mature T and B cells, and set up an adoptive T cell therapy model (Figure 3J). B16F10 melanoma cells stably expressing chicken ovalbumin (OVA) were implanted into Rag1^−/−^ mice, followed by adoptive transfer of OVA‐specific CD8^+^ T cells from OT‐I TCR transgenic mice. We found that, in Rag1^−/−^ mice, *Arhgef3* overexpression failed to inhibit tumor growth (Figure 3K). However, adoptive transfer of OT‐I CD8^+^ T cells restored antitumor immunity and reinstated the growth‐suppressive effect of *Arhgef3* overexpression (Figure 3K), suggesting that CD8^+^ T cells are indispensable mediators of the antitumor immune responses elicited by tumor‐intrinsic ARHGEF3. Collectively, these data indicate that ARHGEF3 promotes a T cell‐inflamed TME characterized by abundant, highly activated, and minimally exhausted T cells, alongside numerically and functionally attenuated MDSCs.

### 2.4 ARHGEF3 Promotes T‐Cell Chemotaxis Via Chemokine Induction

**FIGURE 4:**
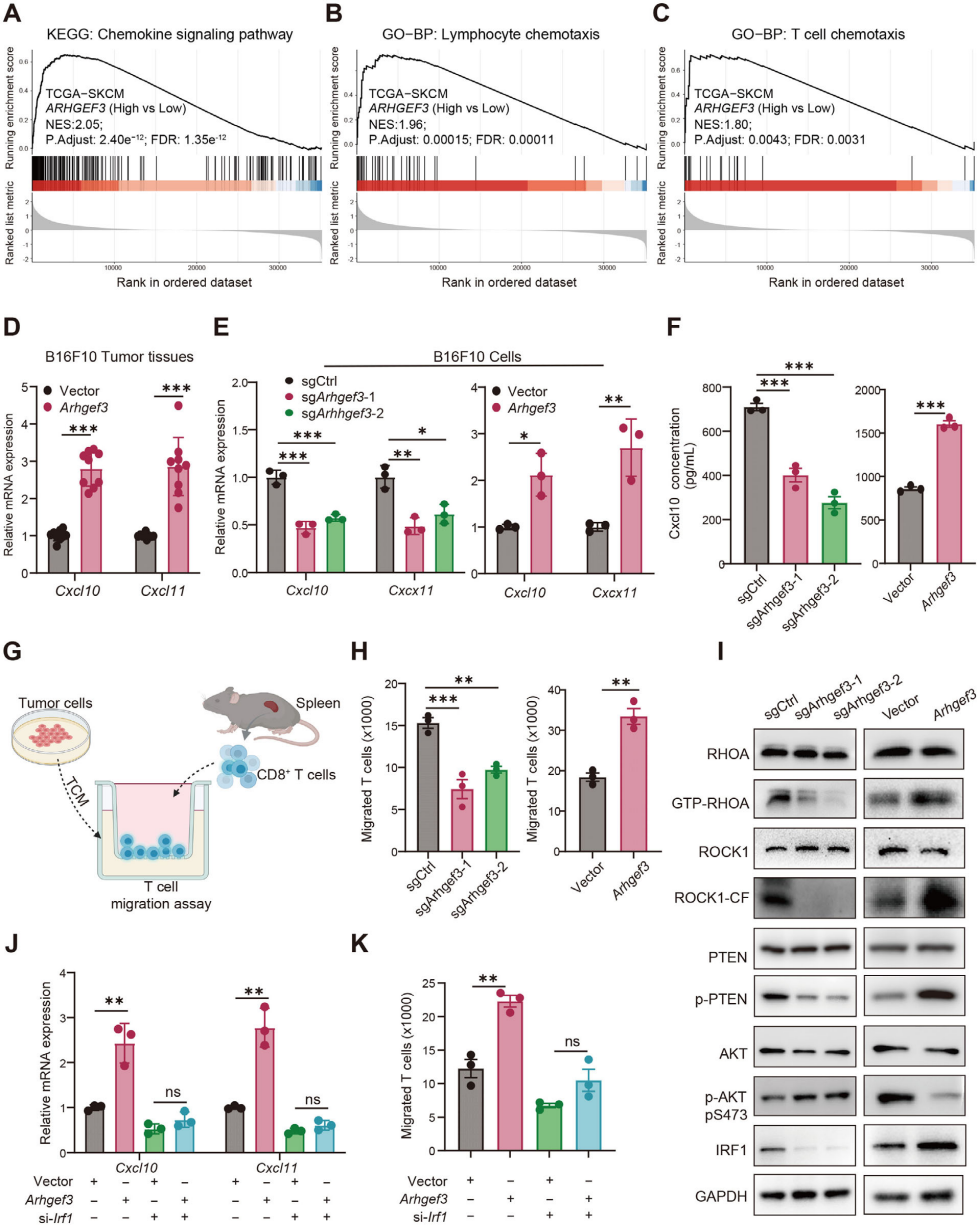
ARHGEF3 promotes chemokine‐dependent T‐cell recruitment via the RHOA–ROCK1–PTEN–AKT–IRF1 axis. (A–C) GSEA analysis of *ARHGEF3*‐high vs. *ARHGEF3*‐low tumors showing enrichment of lymphocyte chemotaxis (A), T‐cell chemotaxis (B), and chemokine‐mediated signaling (C) based on the TCGA‐SKCM cohort. (D) RT‐qPCR analysis of *Cxcl10* and *Cxcl11* in *Arhgef3*‐overexpressing B16F10 tumor tissues. (E) RT‐qPCR analysis of the expression of *Cxcl10* and *Cxcl11* in the indicated B16F10 cells. (F) ELISA quantification of CXCL10 in TCM from the indicated B16F10 cells. (G) Schematic of the T‐cell migration assay; TCM were collected for the T‐cell recruitment assay. (H) Quantification of migrated T cells recruited by TCM from the indicated B16F10 cells. (I) Protein levels along the RHOA‐ROCK1‐PTEN‐AKT‐IRF1 pathway in the indicated B16F10 cells, were determined by western blot. (J,K) After *Irf1* knockdown (si‐*Irf1*) in *Arhgef3*‐overexpressing B16F10 cells, Cxcl10, and Cxcl11 mRNA levels (J) were measured by RT‐qPCR, and T‐cell migration (K) was quantified. *P* value was calculated by an unpaired Student's *t*‐test (*P < 0.05, **P < 0.01, ***P < 0.001). TCM: tumor cells‐derived conditioned medium.

T‐cell infiltration is tightly governed by chemokines in the TME [18]. Across TCGA‐SKCM, ‐LIHC, ‐KIRC, ‐KIRP, and ‐KICH, *ARHGEF3*‐high tumors were enriched for lymphocyte chemotaxis, T‐cell chemotaxis, and chemokine‐mediated signaling (Figure 4A–C, Figure S6A–D), suggesting that ARHGEF3 may promote chemokine‐driven T‐cell recruitment.

To test this idea, we first performed T‐cell‐recruiting chemokine profiling by RT‐qPCR. Among the candidates examined, *Cxcl10* and *Cxcl11* showed the strongest upregulation in *Arhgef3*‐overexpressing cells (Figure S7A). We therefore focused subsequent analyses on these two chemokines. Compared with the control tumors, *Arhgef3*‐overexpressing tumors showed elevated *Cxcl10* and *Cxcl11* expression (Figure 4D). Consistently, flow‐cytometric analysis showed that *Arhgef3*‐overexpressing tumors contained a higher fraction of CXCL10^+^ tumor cells (Figure S7B). At the cellular level, *Arhgef3* overexpression upregulated *Cxcl10*/*Cxcl11* transcripts and enhanced CXCL10 secretion into TCM (Figure 4E,F), whereas *Arhgef3* knockout reduced both mRNA abundance and release (Figure 4E,F, Figure S7C). We further assessed the chemotactic activity of TCM using T‐cell chemotaxis assays (Figure 4G). TCM from *Arhgef3*‐deficient cells attracted fewer T cells (Figure 4H), while TCM from *Arhgef3*‐overexpressing cells significantly enhanced T‐cell migration (Figure 4H). Consistent with these findings, in our Rag1^−^^/^^−^ adoptive T‐cell transfer model (Figure 3K), *Arhgef3*‐overexpressing tumors accumulated more transferred T cells than control tumors (Figure S7D). Together, these data support a positive role for ARHGEF3 in chemokine‐driven recruitment.

AKT inhibition is known to increase CXCL10/CXCL11 through IRF1 [19]. Given that ARHGEF3 negatively regulates AKT, we tested whether ARHGEF3 promotes chemokine expression through the AKT‐IRF1 axis. Consistent with this model, *Arhgef3* knockout increased AKT phosphorylation (p‐AKT) and concomitantly reduced IRF1 (Figure 4I, Figure S7E), whereas *Arhgef3* overexpression lowered p‐AKT and elevated IRF1 (Figure 4I, Figure S7E). Critically, siRNA silencing of IRF1 abolished *Arhgef3*‐dependent induction of *Cxcl10*/*Cxcl11* and eliminated the associated increase in T‐cell migration (Figure 4J,K, Figure S7F). Similarly, CXCR3 blockade largely abolished the *Arhgef3*‐dependent increase in T‐cell chemotaxis in vitro (Figure S7G) and tumor control in vivo (Figure S7H), further supporting that ARHGEF3 promotes T‐cell chemotaxis through the CXCL10/CXCL11–CXCR3 axis.

Because ARHGEF3 is a guanine‐nucleotide exchange factor for RHOA, and RHOA‐ROCK1 signaling is known to activate PTEN, a negative regulator of AKT, we examined this upstream pathway. *Arhgef3* deficiency reduced RHOA activation, decreased ROCK1 activation (including a loss of the proteolytically generated ROCK1‐CF fragment), and downregulated PTEN, culminating in AKT activation (Figure 4I, Figure S7E). Conversely, *Arhgef3* overexpression activated the RHOA‐ROCK1‐PTEN cascade and restrained AKT signaling (Figure 4I, Figure S7E).

Collectively, ARHGEF3 activates a RHOA‐ROCK1‐PTEN brake on AKT, elevating IRF1 to induce CXCL10/CXCL11 in tumor cells. The resulting chemokine gradient enhances T‐cell chemotaxis and supports intratumoral T‐cell accumulation.

### 2.5 ARHGEF3 Suppresses MDSC Accumulation and Function by Inhibiting Fatty Acid Synthesis

To test whether ARHGEF3 regulates MDSCs through mechanisms analogous to those that promote T‐cell infiltration, we first quantified MDSC‐recruiting chemokines, CXCL1 and CXCL2. In B16F10 cells, *Arhgef3* overexpression or knockout did not change *Cxcl1* or *Cxcl2* expression (Figure S8A,B). Consistently, MDSC chemotaxis assays showed that TCM from *Arhgef3*‐overexpressing or ‐deficient cells did not alter MDSC migration (Figure S8C,D). These findings argue against a chemokine‐driven mechanism for MDSC control by ARHGEF3.

**FIGURE 5:**
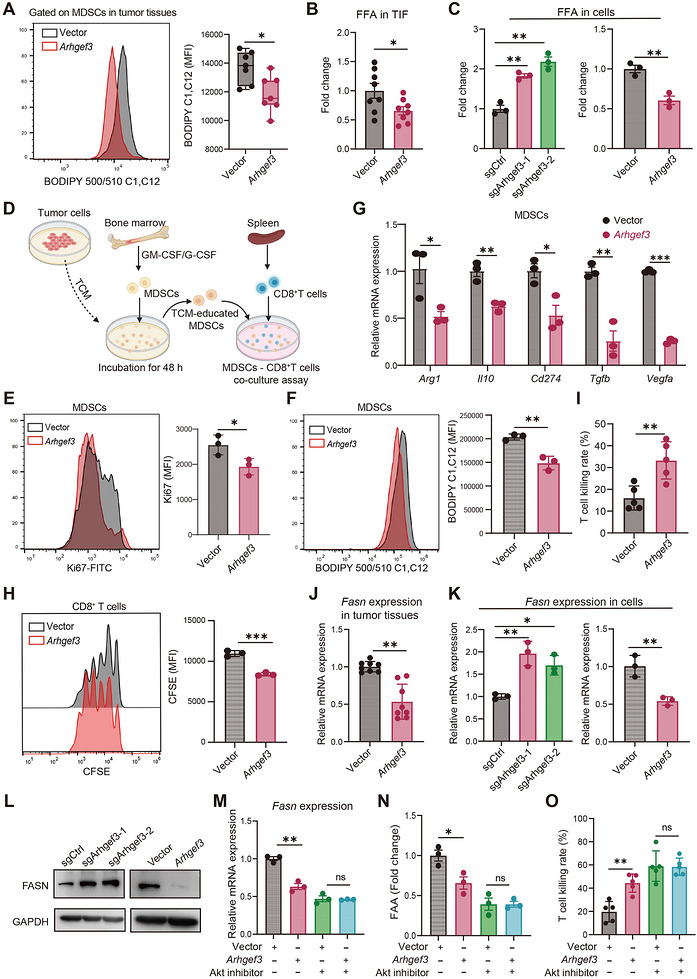
ARHGEF3 limits free‐fatty‐acid production and attenuates the immunosuppressive activity of MDSCs. (A) The content of free fatty acids in intratumoral MDSCs were assessed with BODIPY 500/510 C1, C12 staining. MFI of BODIPY 500/510 C1, C12 was quantified. *n* = 7 mice per group. (B) Quantification of free fatty acids in tumor interstitial fluid. *n* = 8 mice per group. (C) Quantification of free fatty acids in culture supernatants from the indicated B16F10 cells. (D) Schematic of the MDSC‐T‐cell co‐culture assay. Bone marrow‐derived MDSCs were generated by flushing mouse femurs and culturing in medium containing G‐CSF and GM‐CSF. Splenic T cells were isolated and activated with anti‐CD3/CD28 antibodies. BM‐MDSCs were pre‐incubated with TCM for 48 h and then co‐cultured with activated T cells for 48 h. (E) The proliferative capacity of BM‐MDSCs after priming with TCM was assessed by Ki‐67 staining. (F) The content of free fatty acids in BM‐MDSCs after priming with TCM. MFI of BODIPY 500/510 C1, C12 was quantified. (G) RT‐qPCR analysis of the expression of the indicated genes in BM‐MDSCs after priming with TCM. (H) The CFSE level of CD8^+^ T cells after co‐culture with TCM–primed MDSCs, was assessed by flow cytometry; CFSE MFI was quantified. (I) Cytotoxicity of CD8^+^ T cells against tumor cells after prior co‐culture with TCM–primed MDSCs. (J) RT‐qPCR analysis of *Fasn* expression in B16F10 tumor tissues. (K, L) RT–qPCR (K) and immunoblot (L) analysis of FASN expression in the indicated cells. (M‐O) Following treatment of tumor cells with an AKT inhibitor MK2206, *Fasn* expression (M), extracellular free fatty acid levels (N), and T‐cell killing activity (O) were assessed. *P* value was calculated by an unpaired Student's *t*‐test (*P < 0.05, **P < 0.01, ***P < 0.001). MFI: Mean fluorescence intensity; BM‐MDSCs: Bone marrow–‐derived MDSCs; G‐CSF: Granulocyte colony‐stimulating factor; GM‐CSF: Granulocyte‐macrophage colony‐stimulating factor.

We therefore evaluated metabolic support. Because fatty acids sustain MDSC survival and suppressive activity [20], we profiled lipid availability. BODIPY 500/510 staining revealed reduced free‐fatty‐acid accumulation in intratumoral MDSCs from *Arhgef3*‐overexpressing tumors (Figure 5A). Biochemical quantification likewise showed lower free‐fatty‐acid content in *Arhgef3*‐overexpressing tumors (Figure 5B). Conversely, *Arhgef3*‐deficient tumors displayed higher free‐fatty‐acid levels (Figure S9A). In vitro, *Arhgef3* knockout increased free‐fatty‐acid release into TCM (Figure 5C, Figure S9B), whereas *Arhgef3* overexpression suppressed it (Figure 5C). Correspondingly, MDSCs cultured in TCM from *Arhgef3*‐overexpressing cells contained fewer fatty acids, exhibited reduced proliferative capacity, and expressed lower levels of suppressive mediators, including *Arg1*, *Il10*, *Cd274*, *Tgfb*, and *Vegfa* (Figure 5D–G). Functionally, these MDSCs permitted greater T‐cell proliferation and enabled stronger OT‐I T‐cell killing of B16F10‐OVA targets (Figure 5H,I).

Given reports that fatty acids promote macrophage polarization toward an M2‐like state [21], we next asked whether lipid availability also shapes macrophage phenotype in this context. *Arhgef3* overexpression did not alter intratumoral macrophage abundance (Figure 3C,D), but CD206 expression (a M2 marker) on macrophages decreased significantly and the M1 to M2 ratio increased (Figure S10A–C). Immunofluorescence confirmed lower CD206 levels in *Arhgef3*‐overexpressing tissues (Figure S10D), consistent with constrained lipid support for M2‐like polarization.

To define upstream control, we examined the AKT‐FASN axis, which drives fatty acid synthesis. As expected, *Arhgef3*‐overexpressing tumors showed reduced FASN expression (Figure 5J). At the cellular level, *Arhgef3* knockout increased FASN expression at both mRNA and protein levels (Figure 5K,L, Figure S9C), whereas *Arhgef3* overexpression decreased both (Figure 5K,L). Notably, pharmacologic inhibition of AKT abolished *Arhgef3*‐dependent effects on *Fasn* expression and fatty acid release (Figure 5M,N), and eliminated the enhancement of T‐cell cytotoxicity (Figure 5O). To further test the role of FASN, we overexpressed *Fasn* in tumor cells (Figure S9D). *Fasn* overexpression reversed the ARHGEF3‐associated reduction in fatty‐acid levels in TCM (Figure S9E), restored fatty‐acid accumulation in MDSCs (Figure S9F), and rescued MDSC‐mediated suppression of T‐cell cytotoxic activity (Figure S9G).

Taken together, these findings suggest that ARHGEF3 restrains AKT‐FASN‐driven fatty‐acid synthesis, thereby limiting MDSC‐mediated immunosuppression and supporting effective T‐cell immunity.

### 2.6 ARHGEF3 Sensitizes Tumors to Anti‐PD‐1 Immunotherapy

**FIGURE 6:**
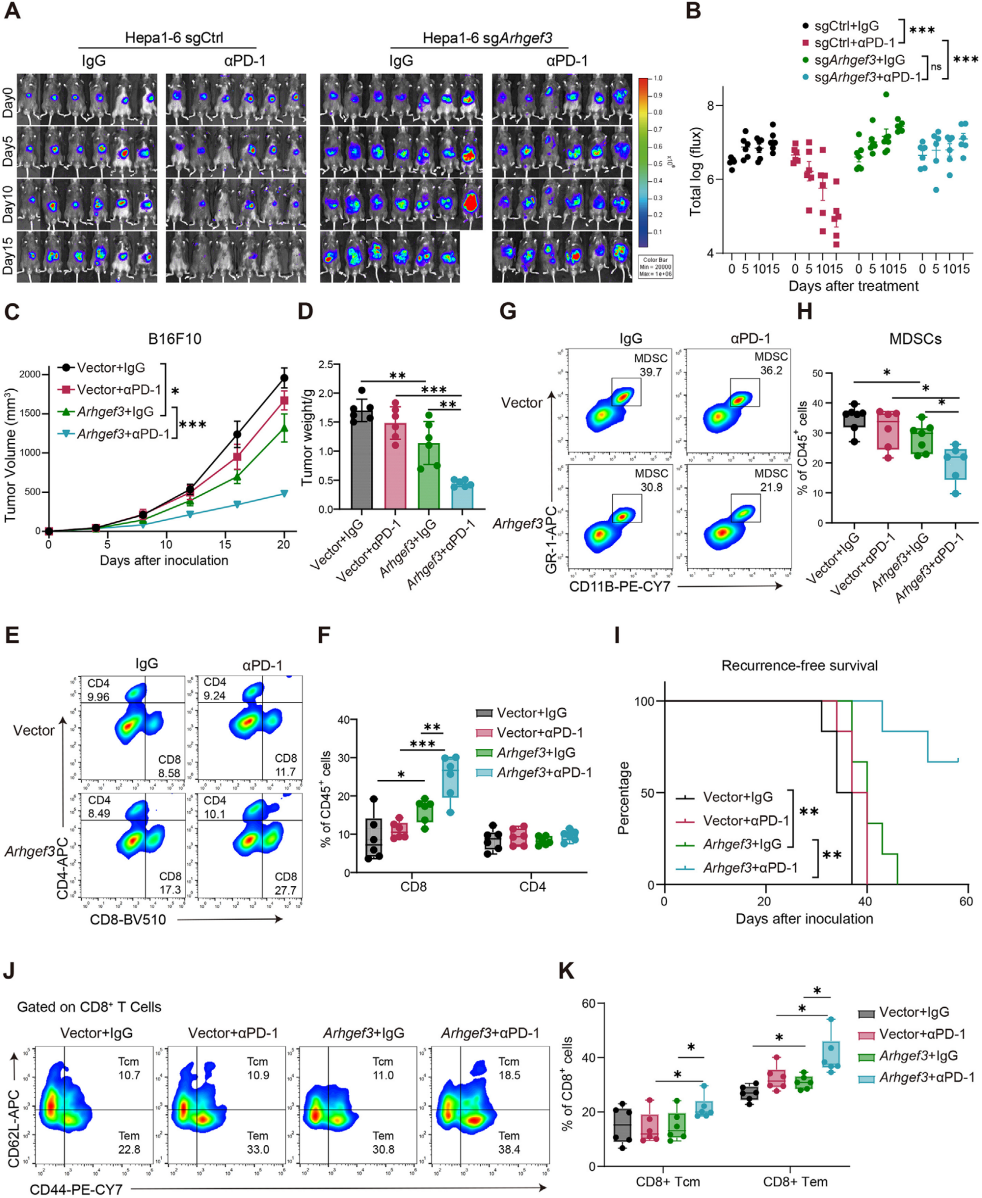
ARHGEF3 re‐sensitizes tumors to anti–PD‐1 immunotherapy. (A) Representative bioluminescence images of mice bearing orthotopic *Arhgef3*‐sgRNA Hepa1‐6 tumor after antiPD‐1 treatment. n=6 mice per group. (B) Quantification of tumor bioluminescence of *Arhgef3*‐sgRNA Hepa1‐6 tumors. (C,D) Tumor growth curves (C) and endpoint tumor weights (D) of *Arhgef3‐*overexpressing B16F10 tumors following anti‐PD‐1 treatment. *n* = 6 mice per group. (E,F) Representative flow cytometry plots (E) and quantification (F) of intratumoral CD8^+^ and CD4^+^ T‐cell frequencies in B16F10 tumors after indicated treatment. (G,H) Representative flow cytometry plots (G) and quantification (H) of intratumoral MDSC frequencies in B16F10 tumors after indicated treatment. (I) Recurrence‐free survival of B16F10 tumor‐bearing mice after indicated treatment. *n* = 6 mice per group. (J,K) Representative flow cytometry plots (J) and quantification (K) of central memory (Tcm) and effector memory (Tem) T‐cell subsets in B16F10 tumors after anti–PD‐1 treatment. *P* values were calculated by unpaired Student's *t*‐test, two‐way ANOVA, or the log‐rank (Mantel‐Cox) test (*P < 0.05, **P < 0.01, ***P < 0.001).

Given the central role of the tumor immune microenvironment in shaping immunotherapy outcomes, we asked whether ARHGEF3 modulates responses to ICB. We employed two complementary models: an immunotherapy‐sensitive orthotopic Hepa1‐6 hepatocellular carcinoma model and an immunotherapy‐resistant subcutaneous B16F10‐OVA melanoma model. In the Hepa1‐6 model, anti‐PD‐1 conferred a clear therapeutic benefit (Figure 6A,B), which was significantly attenuated by *Arhgef3* knockout (Figure 6A,B), indicating that endogenous ARHGEF3 supports ICB efficacy. In the B16F10‐OVA model, anti‐PD‐1 monotherapy showed little therapeutic effect (Figure 6C,D), consistent with intrinsic resistance, whereas *Arhgef3* overexpression overcame this resistance and markedly improved anti‐PD‐1 efficacy (Figure 6C,D).

We further determined the immune basis of these effects. *Arhgef3* overexpression increased intratumoral CD8^+^ cytotoxic T‐cell infiltration and function, while reducing MDSC accumulation (Figure 6E–H, Figure S11A). Combination with anti‐PD‐1 further amplified these changes (Figure 6E–H, Figure S11A), in line with the observed suppression of tumor growth. Moreover, depletion of CD8^+^ T cells completely abrogated the therapeutic benefit of the combination treatment (Figure S11B), demonstrating that this sensitizing effect is CD8^+^ T‐cell dependent. We next asked whether the combination promotes durable immunity. As expected, the combination provided the strongest control of tumor recurrence and delayed the growth of re‐challenged tumors (Figure 6I, Figure S11C). Tumor relapses are typically related to re‐expansion of memory T‐cell populations [22], which are subdivided into CD44^+^CD62L^+^ central memory T cells and CD44^+^CD62L^−^ effector memory T cells [23]. To explore the potential for ARHGEF3‐induced expansion of memory T cells in vivo, the levels of Tem and Tcm (gated on CD8^+^ and CD4^+^ T cells) were determined in the spleen of mice at the beginning of tumor relapse. As expected, Mice bearing *Arhgef3*‐overexpressing tumors and treated with anti‐PD‐1 therapy displayed higher frequencies of both Tem and Tcm than mice receiving anti‐PD‐1 alone (Figure 6J,K, Figure S11D,E). These findings indicate that ARHGEF3 strengthens systemic antitumor immunity during PD‐1 blockade and prevents tumor relapse.

### 2.7 Human ARHGEF3 Expression Marks an Immunoreactive TME and Predicts Immunotherapy Benefit

**FIGURE 7:**
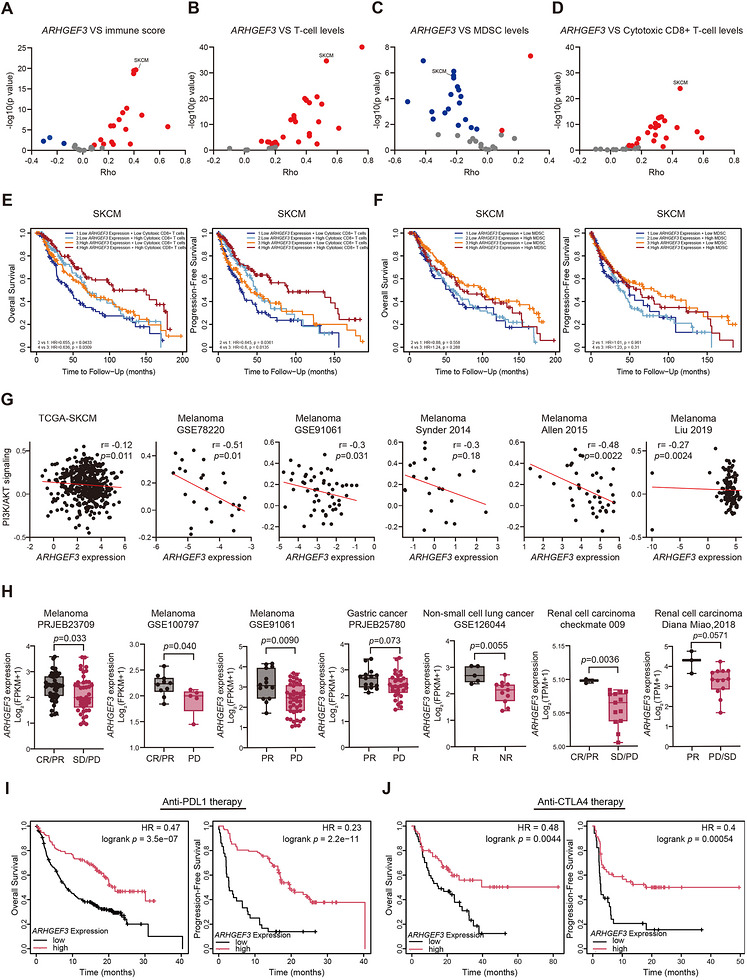
Human ARHGEF3 expression associates with a T cell–inflamed tumor microenvironment and immunotherapy outcomes. (A–D) Pan‐cancer correlations between *ARHGEF3* expression and immune score (A), T‐cell infiltration (B), MDSC abundance (C), and cytotoxic CD8^+^ T‐cell signature (D) in the TCGA database. (E) Overall survival (left) and progression‐free survival (right) in TCGA‐SKCM, stratified jointly by *ARHGEF3* expression and the cytotoxic CD8^+^ T‐cell signature. (F) Overall survival and progression‐free survival in TCGA‐SKCM, stratified jointly by *ARHGEF3* expression and MDSC level. (G) Correlation of *ARHGEF3* expression with PI3K‐AKT oncogenic signaling in TCGA‐SKCM and additional melanoma cohorts. *P* values were calculated by Spearman's rank correlation. (H) *ARHGEF3* expression in responder vs non‐responder across multiple immunotherapy datasets (response categories: CR, complete response; PR, partial response; SD, stable disease; PD, progressive disease). *P* values were calculated by the Mann‐Whitney U test. (I,J) Overall survival (left) and progression‐free survival (right) in anti‐PD‐L1 (I) and anti‐CTLA‐4 (J) cohorts stratified by *ARHGEF3* expression. *P* values were calculated by the log‐rank (Mantel‐Cox) test.

We next extended these findings to human cancers. First, we explored immune cell infiltration according to the ESTIMATE algorithm, and found that ARHGEF3 expression was positively correlated with the immune score in most cancer types (Figure 7A). To more specifically capture T‐cell infiltration, we applied a T‐cell infiltration signature based on 15 T cell‐restricted genes (*CD2, CD247, CD28, CD3D, CD3G, CD6, GPR171, GZMK, ICOS, ITK, KLRB1, PYHIN1, TIGIT, TRAT1, TRBC1*) [24]. *ARHGEF3* expression was positively correlated with this T‐cell infiltration signature across cancer types (Figure 7B), indicating a tighter association with intratumoral T cell presence. Consistent with a chemokine‐driven mechanism, *ARHGEF3* expression was also correlated positively with the expression of *CXCL10*, *CXCL11*, the receptor *CXCR3*, and the transcription factor *IRF1* in most cohorts (Figure S12A–D), supporting involvement of the IRF1‐CXCL10/11‐CXCR3 axis in T‐cell recruitment. In line with this, our bladder urothelial carcinoma cohort showed that *ARHGEF3*‐high tumors displayed elevated *CD8A* expression (Figure S12E). In contrast, *ARHGEF3* expression was correlated negatively with the abundance of MDSCs and with M2‐like macrophages across most cancer types (Figure 7C, Figure S12F). Notably, these negative associations did not extend to *CXCL1* or *CXCL2* (figure S12G,H), arguing against a chemotactic explanation for lower MDSC levels. To relate infiltration to effector function, we next leveraged a set of five reported genes (*CD8A*, *CD8B*, *GZMA*, *GZMB*, and *PRF1*) to define a cytotoxic T lymphocyte (CTL) score [25]. ARHGEF3 expression was correlated positively with the CTL score (Figure 7D) as well as with additional T cell‐functional markers, including *CD69*, *CD137*, and *TCF7* (Figure S12I–K). Finally, in human melanoma tissues, although ARHGEF3 staining was generally low, the ARHGEF3‐positive area within tumor regions was positively correlated with CD8^+^ T‐cell density (Figure S13), providing histologic support for the association between ARHGEF3 expression and T‐cell infiltration. Together, these data indicate that high *ARHGEF3* expression marks an immunoreactive tumor microenvironment characterized by enhanced T‐cell infiltration and reduced immunosuppressive myeloid populations.

Among all cancer types, these associations were most pronounced in SKCM. Within SKCM, patients with high *ARHGEF3* and high CD8 T‐cell levels had the best overall survival and progression‐free survival, whereas those with low *ARHGEF3* and low CD8^+^ T‐cell levels had the worst outcomes (Figure 7E). Similarly, high *ARHGEF3* together with low MDSC abundance defined a favorable‐prognosis subgroup (Figure 7F). These stratifications underscore the alignment of *ARHGEF3* with a T cell‐inflamed, immunoreactive tumor microenvironment. To test the tumor‐intrinsic basis for this immune phenotype, we next examined PI3K‐AKT signaling in human melanoma. In TCGA‐SKCM, ARHGEF3 expression was negatively correlated with PI3K‐AKT and downstream mTORC1 signaling (Figure 7G, Figure S12L). This association was observed in multiple independent melanoma cohorts (Figure 7G, Figure S12L). *ARHGEF3* expression was also negatively correlated with *FASN* expression (Figure S12M), consistent with an AKT‐FASN axis in lipid control.

Finally, we evaluated potential clinical relevance for immunotherapy. The T cell‐inflamed gene‐expression profile (GEP) and tertiary lymphoid structures (TLS), both linked to reduced immune evasion and higher likelihood of ICB response [26, 27], were positively correlated with *ARHGEF3* expression in most tumor types (Figure S12N). Consistently, across multiple independent cohorts of patients treated with immunotherapy, responders exhibited higher *ARHGEF3* expression than non‐responders (Figure 7H). Pan‐cancer survival analyses of ICB‐treated patients further showed that high *ARHGEF3* expression was associated with longer overall and progression‐free survival (Figure 7I,J, Figure S12O). Together, these results align with our experimental data and support a link between ARHGEF3 and enhanced responsiveness to ICB.

Taken together, multi‐cohort correlations and clinical associations indicate that ARHGEF3 marks an immunoreactive, T cell‐inflamed tumor state and may help identify patients more likely to benefit from ICB.

## 3 Discussion

**FIGURE 8:**
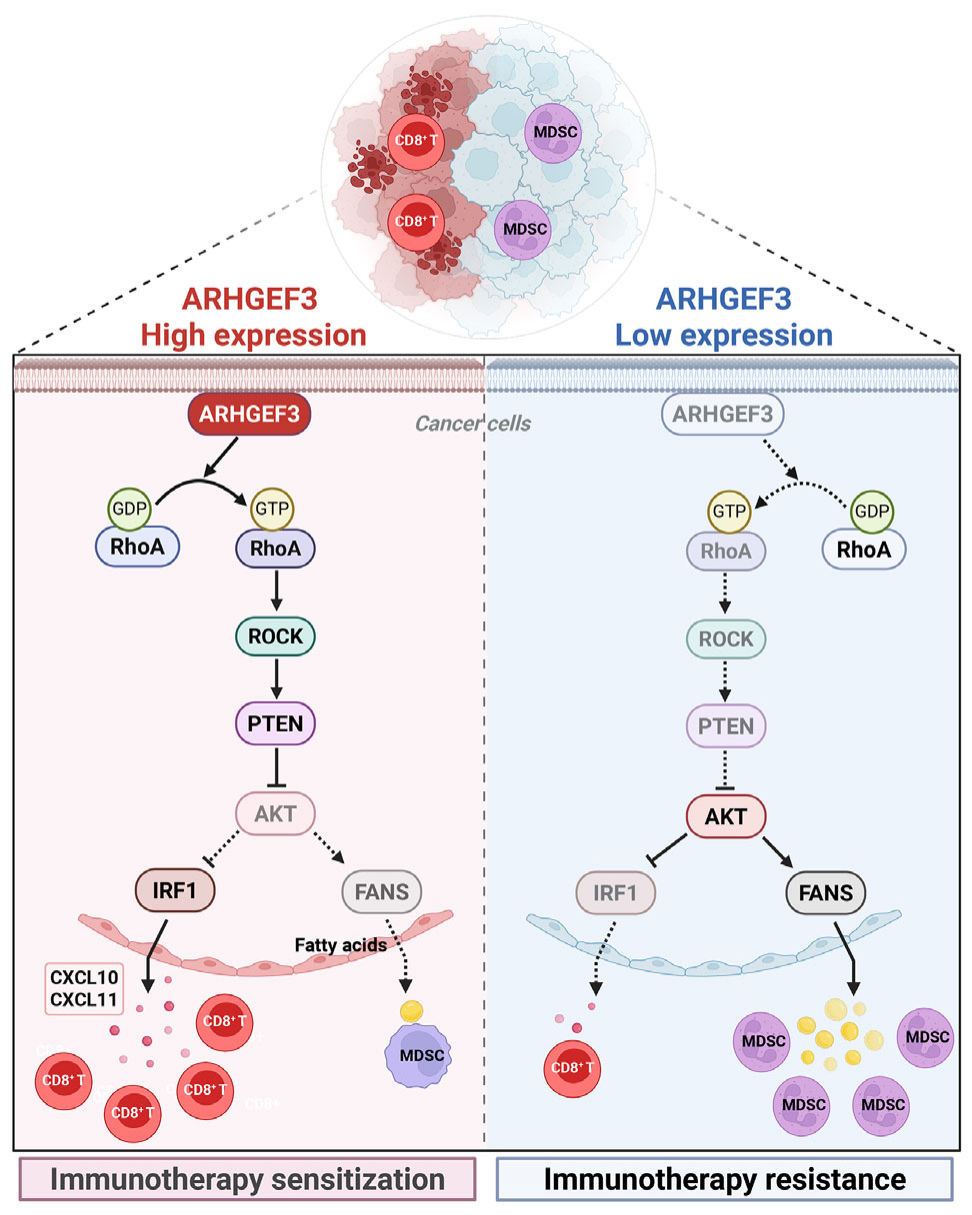
Schematic depiction of the dual role of ARHGEF3 in promoting T‐cell infiltration and relieving MDSC‐mediated immunosuppression.

Immunotherapy has reshaped cancer treatment, yet a substantial proportion of patients do not respond or derive only transient benefit. A pragmatic route to broaden efficacy is to pair T cell–directed interventions with strategies that recondition myeloid compartments. Mechanistically, the immunologically “cold” phenotype of many solid tumors often reflects two convergent defects: shallow chemokine gradients that limit T‐cell entry, and dominant suppressive myeloid networks that further constrain cytotoxic responses [28, 29, 30]. Against this backdrop, we show that a tumor‐intrinsic regulator can simultaneously enhance T‐cell access and relieve myeloid cell‐mediated suppression, thereby converting a non‐responsive microenvironment into one more permissive for durable control (Figure 8).

Members of the ARHGEF family exhibit marked context dependence in cancer biology. For example, activation of ARHGEF10 restrains pancreatic cancer progression [31], whereas its homolog ARHGEF10‐like (ARHGEF10L) promotes hepatocarcinogenesis [32]; ARHGEF12 suppresses tumorigenesis in colorectal and breast cancers but enhances tumorigenicity in neuroblastoma [33, 34]. Reports on ARHGEF3 are likewise mixed: it has been described to promote proliferation in nasopharyngeal and lung cancer cells [35, 36], yet to inhibit invasion, metastasis, and proliferation in osteosarcoma [37]. In our study, using the Hepa1‐6 hepatocellular carcinoma, B16F10 melanoma, and Renca renal cell carcinoma models, *Arhgef3* overexpression or CRISPR–Cas9 knockout did not produce obvious changes in tumor‐cell proliferation in either in vitro cell systems or in vivo athymic nude‐mouse models. Consistent results were obtained with two independent sgRNAs targeting *Arhgef3*. These observations suggest that previously reported proliferative effects more likely reflect lineage‐specific network wiring rather than a uniform cell‐autonomous program.

RHOA likewise exhibits duality. In some contexts, RHOA activation promotes bladder‐cancer proliferation and invasion via ROCK/YAP [38]; in others, RHOA limits colorectal‐cancer proliferation, invasion, and de‐differentiation by suppressing Wnt/β‐catenin signaling [39]. These observations support the view that the net outcome of RHOA signaling is jointly determined by upstream ARHGEF members together with cellular context, signaling dose, and pathway wiring. Within this landscape, ARHGEF3 appears to channel RHOA output toward immune‐supportive programs in vivo rather than simply regulating cell proliferation. Mechanistically, the ROCK/PTEN branch provides a route by which RHOA restrains oncogenic AKT signaling. Prior work shows that loss of RHOA function can dampen ROCK1/PTEN signaling, elevate AKT activity, and foster an immunosuppressive TME [19]. Consistent with this, in our models, ARHGEF3 promotes immune reprogramming through an RHOA/ROCK1/PTEN axis. It relieves AKT‐mediated repression of IRF1 to enhance T‐cell infiltration, and suppresses the AKT/FASN axis to limit fatty‐acid synthesis, thereby reducing activation of MDSCs. This dual immune‐enhancing effect mediated by AKT inhibition ultimately induces strong T‐cell immunity and suppresses tumor progression.

Chemokines play critical roles in T‐cell trafficking, which prompted us to investigate the chemokine‐centric regulatory modules responsible for the recruitment of T cells. CXCL9, CXCL10, and CXCL11 are commonly linked to intratumoral T‐cell infiltration via their shared receptor CXCR3 [40]. In our experiments, chemokine profiling together with T‐cell migration assays showed that ARHGEF3 promotes T‐cell recruitment primarily through CXCL10 and CXCL11. Consistent with this, CXCR3 blockade markedly reduced T‐cell chemotaxis and largely abrogated the enhanced antitumor efficacy observed in *Arhgef3*‐overexpressing tumors. In addition, although the overall abundance of CD4^+^ T cells changed little, Th1‐like CD4^+^ T cells increased (data not shown), indicating selective remodeling within the CD4^+^ compartment.

Tumors commonly reprogram metabolism to shape antitumor immunity, and lipid metabolism is a major driver of immunosuppression in the TME. Myeloid populations preferentially use fatty acids to acquire and maintain suppressive phenotypes. Distinct from its chemokine‐based promotion of T‐cell infiltration, ARHGEF3 appears to regulate the myeloid compartment through lipid metabolism. We found that ARHGEF3 lowers fatty‐acid release from tumor cells and reduces fatty‐acid accumulation within intratumoral MDSCs. Although the precise lipid species remain to be defined, convergence on the AKT‐FASN axis provides a coherent upstream explanation and a tractable pharmacologic node. Further definition of the relevant lipid species, together with testing whether their replenishment can restore ARHGEF3‐dependent phenotypes, should help clarify this pathway in greater mechanistic detail. Beyond MDSCs, fatty‐acids have been reported to promote tumor‐associated macrophage polarization toward M2‐like states. Consistent with this, total macrophage abundance changed little in our systems, yet ARHGEF3 shifted polarization, moving the M1/M2 balance toward an antitumor phenotype. Fatty‐acid metabolism may also influence other immune populations, including DCs and NK cells. Although we did not observe obvious changes in the proportions of NK cells or DCs in our experiments, we cannot exclude the possibility that ARHGEF3 also modulates their functional state by reducing fatty‐acid availability in the TME, which will require further investigation. Together, these data support a model in which ARHGEF3 reshapes the composition and functional state of the myeloid compartment primarily by restricting metabolic supply rather than by altering chemokine‐driven recruitment.

The ideal therapeutic strategy simultaneously enhances T‐cell abundance and restores T‐cell function. Beyond promoting chemokine‐guided T‐cell entry, ARHGEF3 reduces the suppressive activity of MDSCs and M2‐like macrophages, thus lifting the brakes on T‐cell cytotoxicity and enabling better responses to ICB. Across clinical datasets, *ARHGEF3* is downregulated in many tumor types. Higher *ARHGEF3* associates positively with T‐cell infiltration/activation signatures and inversely with MDSC and M2‐macrophage signatures. This effect is most pronounced in melanoma. In immunotherapy cohorts, patients with *ARHGEF3*‐high expression show stronger response rates and longer survival. Notably, the current findings support a primarily tumor‐cell‐intrinsic role of ARHGEF3 in regulating the TME, but do not address whether host‐derived *Arhgef3* also contributes to this process. Future studies using *Arhgef3* genetic mouse models, including bone marrow chimera approaches, will be needed to clarify this question.

Although no specific ARHGEF3 agonist is currently available for clinical use, our findings suggest that therapeutic activation of ARHGEF3 may represent a promising strategy. Because the antitumor effects of RHOA activation are likely shaped by upstream signaling context, direct activation of RHOA alone may be insufficient or suboptimal. Thus, future development of specific ARHGEF3 agonists will be important for translating these findings into therapeutic applications. Taken together, our work supports the central conclusion that ARHGEF3 is a tumor‐intrinsic, immune‐activating determinant with potential to enhance the efficacy of cancer immunotherapy.

## 4 Experimental Section

### 4.1 Mice and Tumor Cell Lines

Male C57BL/6 and BALB/c mice (4–6 weeks old) were purchased from Shanghai Lingchang Biotechnology or Jiangsu Jicui Pharmaceutical Technology and housed under specific pathogen‐free conditions. All animal procedures were approved by the Ethics Committee of Shanghai University of Traditional Chinese Medicine (No. SYXK 2020‐0014). Murine tumor cell lines B16‐F10 (CVCL_0159), Hepa1‐6 (RRID: CVCL_0327), and Renca (CVCL_2174) were obtained from the American Type Culture Collection (ATCC) or the Cell Bank of the Shanghai Institutes for Biological Sciences, Chinese Academy of Sciences (SIBS, CAS). The B16F10‐OVA line was generated by lentiviral transduction with an ovalbumin (OVA) expression construct, as previously described [41]. Cells were cultured in DMEM or RPMI‐1640 supplemented with 10% fetal bovine serum, 100 U/mL penicillin, and 100 µg/mL streptomycin. All cell lines used in this study were routinely tested and confirmed to be mycoplasma‐free.

### 4.2 Reagents and Antibodies

Anti‐PD‐1 antibody (clone: RMP1‐14) and isotype control were obtained from BioXcell. The fluorochrome‐labeled anti‐mouse antibodies used for flow cytometry were Fixable Viability Stain 780 (565388; BD Biosciences); PE‐CD45 (553081; BD Biosciences); FITC‐CD45 (553080; BD Biosciences); BV510‐CD45 (563891; BD Biosciences); FITC‐CD3e (553061; BD Biosciences); BV421‐CD8 (563898; BD Biosciences); BV510‐CD8 (563068; BD Biosciences); APC‐CD4 (553051; BD Biosciences); FITC‐CD4 (553080; BD Biosciences); PE‐Cy7‐CD11b (552850; BD Biosciences); FITC‐Ly6G (551460; BD Biosciences); APC‐Ly6C (560595; BD Biosciences); BV421‐F4/80 (123132; Biolegend); PerCP‐Cy5.5‐I‐A/I‐E (562363; BD Biosciences); PE‐NK‐1.1 (553165; BD Biosciences); APC‐IFN‐γ (554413; BD Biosciences); PE‐TNF‐α (506306;Biolegend);CD16/32 (553141; BD Biosciences); APC‐CD206 (141707; Biolegend). The anti‐mouse antibodies used for immunobloting were Anti‐ARHGEF3 antibody (A8490; Abclonal); anti‐RHOA antibody (S0B1253; STARTER); anti‐ROCK1 antibody (S0B1040; STARTER); anti‐PTEN antibody (S0B2106; STARTER); anti‐phospho‐PTEN antibody (9551; CST); anti‐IRF1 antibody (11335‐1‐AP; Proteintech); anti‐AKT antibody (4691; CST); anti‐phospho‐AKT antibody (4060; CST); anti‐FASN antibody (S0B1253; STARTER); anti‐GAPDH antibody (2118; CST); Anti‐rabbit IgG antibody (5127; CST).

### 4.3 Gene Knockout and Overexpression

Arhgef3‐knockout tumor cell line was generated using the lentiCRISPRv2 system to deliver single‐guide RNAs (sgRNAs) targeting the Arhgef3 coding sequence. Knockout was confirmed by western blot. The sgRNA sequences used were: control sgRNA, 5′‐GCGAGGTATTCGGCTCCGCG‐3′; Arhgef3‐sgRNA‐1, 5′‐TGTCGGGGCGGCTCTCGCTG‐3′; Arhgef3‐sgRNA‐2, 5′‐CTGATTGACACACACATCGA‐3′; Arhgef3‐sgRNA‐3, 5′‐GCGCTTCAGCCAGACCTTGC‐3′. For overexpression, full‐length ARHGEF3 was cloned into a lentiviral expression vector and introduced into cells; expression was confirmed by western blot.

### 4.4 In Vivo Tumor Models

For subcutaneous tumor models, Hepa1‐6 (1.5×10^6^) or B16F10 (2.0×10^5^) cells were injected subcutaneously into the left flank of C57BL/6 mice, and Renca (1.0×10^6^) cells were injected subcutaneously into the left flank of BALB/C mice. Tumor volume was calculated by the formula of length×width^2^/2. When tumors reached approximately 5 mm in diameter, mice were randomized and subjected to the indicated treatments. Mice were euthanized when tumor volume exceeded 2000 mm^3^, in accordance with institutional ethical guidelines. For the luciferase‐labeled orthotopic liver tumor model, 1.0×10^6^ Hepa1‐6 cells stably expressing firefly luciferase (1.0 × 10^6 in 100 µL medium) were orthotopically injected into the liver of C57BL/6 mice under anesthesia. Tumor growth was monitored by bioluminescence imaging using an IVIS system (PerkinElmer, Fremont, CA, USA) every 5 days starting on day 8 post‐implantation. For the adoptive T‐cell therapy model, B16F10‐OVA cells were implanted subcutaneously into the left flank of Rag1^−/−^ mice. OT‐I CD8^+^ T cells were isolated from the spleens of OT‐I mice using the EasySep Mouse CD8^+^ T Cell Isolation Kit (Stemcell). When tumors reached ∼500 mm^3^, activated OT‐I CD8^+^ T cells (8×10^5^) were adoptively transferred intravenously. To block the PD‐L1/PD‐1 signaling pathway, tumor‐bearing mice were injected intraperitoneally with anti‐PD‐1 antibodies (100 µg per mouse) every 4 days. To establish the tumor rechallenge model, B16F10 tumor cells (2.0×10^5^) were implanted subcutaneously into the left flank of C57BL/6 mice. Mice received anti‐PD‐1 antibody treatment, and the primary tumors were resected on day 12 post‐implantation. After two weeks recovery period, B16F10 cells (4.0×10^5^) were re‐inoculated subcutaneously into the contralateral flank. Tumor growth was then monitored.

### 4.5 RNA Extraction and Quantitative Real‐Time PCR

Total RNA was extracted using TRIzol (Invitrogen) and reverse‐transcribed into cDNA. Quantitative real‐time PCR (RT‐qPCR) was performed on an ABI QuantStudio Dx system (Applied Biosystems). Target gene expression was normalized to β‐actin. Primer sequences are listed in Table S1.

### 4.6 Western Blot

Tumor cells were lysed on ice in RIPA buffer (PC101; Epizyme) supplemented with freshly added protease inhibitor cocktail (5872; CST). After 20 min, cell lysates were centrifuged at 12 000 rpm for 15 min at 4°C, and protein concentrations in the supernatant were determined by Pierce BCA Protein Assay Kit (23 225; Thermo Fisher Scientific). Equal amounts of protein were mixed with 6× SDS loading buffer (DL101; TransGen), heated at 95°C for 10 min, separated by SDS‐PAGE, and transferred to PVDF membranes (ISEQ00010; Millipore). Membranes were blocked in Tris‐buffered saline plus 0.1% Tween 20 (TBST) containing 5% nonfat dry milk for 1 h and probed with the indicated antibodies overnight. Membranes were washed with TBST for three times and incubated with HRP‐conjugated secondary antibodies for 2 h. The bands were detected with the Tanon‐5200 Chemiluminescent Imaging System (Tanon Science & Technology).

### 4.7 Analysis of Infiltrating Immune Cells by Flow Cytometry

Mice were euthanized, and tumors were excised, minced, and dissociated into single‐cell suspensions using a Mouse Tumor Dissociation Kit (Miltenyi Biotec) per the manufacturer's instructions. Cell suspensions were filtering through a 70‐µm Falcon cell strainer (Corning), blocked with anti‐mouse CD16/32 (Fc block) for 15 min, and stained with Fixable Viability Stain 780 for 10 min to exclude dead cells. After washing, cells were incubated with the indicated surface antibodies for 30 min in the dark, washed twice with staining buffer, and analyzed on a CytoFLEX LX flow cytometer (Beckman Coulter). For intracellular cytokine staining, tumor‐infiltrating leukocytes were enriched by OptiPrep (Sigma) density gradient centrifugation and restimulated at 37 °C with Leukocyte Activation Cocktail plus BD GolgiPlug (BD Biosciences) for 4–6 h, followed by fixation and permeabilization with the Cytofix/Cytoperm kit (BD Biosciences). For intranuclear staining, cells were processed with the Foxp3/Transcription Factor Staining Buffer Set (eBioscience) and stained as described above. For assessment of fatty acid content, intratumoral MDSCs were incubated with BODIPY 500/510 C1, C12 (Beyotime Biotechnology) for 30 min and subjected to flow cytometry.

### 4.8 Isolation of Tumor Interstitial Fluid and Free Fatty Acid Analysis

Tumor interstitial fluid was collected from excised tumors. Briefly, intact tumor tissue was washed with phosphate‐buffered saline, blotted dry, and placed on a 70‐µm Falcon cell strainer (Corning) installed in 50 mL centrifuge tubes. and cut into pieces. Samples were centrifuged at 400 g for 15 min at 4°C. The supernatant was collected and further centrifuged at 10 000 g for 5 min at 4°C. The final supernatant was used for lipid measurements. Total free fatty acids were quantified using the Amplex Red Free Fatty Acid Assay Kit (Beyotime Biotechnology) according to the manufacturer's instructions.

### 4.9 T Cell Co‐Culture Assay

Mouse splenic CD8^+^ T cells were isolated using the EasySep Mouse CD8^+^ T‐cell Isolation Kit (STEMCELL Technologies) according to the manufacturer's instructions. For the CD8^+^ T cell proliferation assay, purified CD8^+^ T cells were labeled with CFSE using the CellTrace CFSE Cell Proliferation Kit (Invitrogen). Labeled CD8^+^ T cells were co‐cultured with tumor cell‐derived conditioned medium (TCM)‐educated MDSCs at a 1:1 ratio in the plate pre‐coated with anti‐CD3 (1 µg/mL; eBioscience) and anti‐CD28 (2 µg/mL; eBioscience) for 48 h. Cells were harvested, and the intensity of the CFSE signal in the gated CD8^+^ T cells was measured by flow cytometry. For the T cell killing assay, OT‐I T cells were isolated from OT‐I transgenic mice, activated, and co‐cultured with TCM‐educated MDSCs for 48 h. Then OT‐I T cells were co‐cultured with B16F10‐OVA target cells for 24 h. Cells were harvested and counted. The proportion of residual tumor cells, represented as CD45^−^ events, was measured by flow cytometry.

### 4.10 Cell Migration Assay

CD8^+^ T cells were isolated from the spleens of C57BL/6 mice, and activated with anti‐CD3/CD28 antibody for 48 h. Bone marrow–derived MDSCs (BM‐MDSCs) were obtained by flushing the femurs of mice and cultured for 6 days in RPMI‐1640 supplemented with 10% FBS, 100 U/mL penicillin, 100 µg/mL streptomycin, and 10 ng/mL each of GM‐CSF and G‐CSF. For migration assays, 1×10^6^ CD8^+^ T cells or BM‐MDSCs in complete media were loaded into the upper chamber of Transwell inserts (5.0 µm pore size; Corning). The lower chamber was filled with TCM. Plates were incubated for 4–6 h, after which cells in the lower chamber were collected and counted. For CXCR3 blockade, CD8^+^ T cells were pretreated with the CXCR3 inhibitor SCH546738 (20 nM; TargetMol) for 1 h before being added to the upper chamber.

### 4.11 Immunofluorescence Staining

Paraffin‐embedded tissues were sectioned at 4 µm, deparaffinized, rehydrated, and subjected to citrate‐based antigen retrieval. After blocking, sections were incubated overnight at 4°C with primary antibodies against CD8α, GR‐1 (Ly6G/Ly6C), and CD206. After PBS washes, species‐specific Alexa Fluor‐conjugated secondary antibodies were applied for 1 h in the dark. Nuclei were counterstained with DAPI in an anti‐fade mounting medium. Images were acquired on a Leica TCS SP8 confocal microscope. At least 5 fields were collected for each sample. All primary and secondary antibodies were from Servicebio (Wuhan, China).

### 4.12 Database Analysis

TCGA pan‐cancer gene‐expression and clinical data were downloaded from the NCI Genomic Data Commons (GDC) portal (https://portal.gdc.cancer.gov). Gene‐expression and clinical data for GSE100797, GSE91061, GSE126044, PRJEB23709, and PRJEB25780 were downloaded from the GEO or TIGER database (http://tiger.canceromics.org). Correlations between ARHGEF3 and the immune score, MDSC levels, and M2‐like macrophage levels were evaluated using the TIMER3 database (https://compbio.cn/timer3). Correlations between ARHGEF3 and oncogenic PI3K‐AKT and mTORC1 signaling were assessed using the IMPACT database (http://www.brimpact.cn). Correlations between ARHGEF3 and the T cell‐inflamed gene‐expression profile (GEP) and tertiary lymphoid structure (TLS) signatures using the TIGER database. Overall survival and progression‐free survival according to ARHGEF3 expression were analyzed in Pan‐cancer and in Immunotherapy cohorts using the Kaplan–Meier Plotter database (https://kmplot.com). Gene set enrichment analysis (GSEA) of TCGA‐SKCM, LIHC, KIRC, KIRP, and KICH was performed in R using the clusterProfiler package. The single‐cell expression levels of ARHGEF3 across various tumor tissues were analyzed using the TISCH2 database (http://tisch.compbio.cn/home).

### 4.13 Clinical Samples

A total of 18 bladder cancer tissue samples (15 paired paratumor tissues) and 13 renal cell carcinoma tissue samples (12 paired paratumor tissues) were obtained from the Fudan Shanghai Cancer Center. The study including use of tissues and associated clinical data, was approved by the Institutional Review Board of Fudan Shanghai Cancer Center (No. 050432‐4‐2108). Six paired lung adenocarcinoma tissue samples were obtained from Shanghai Municipal Hospital of Traditional Chinese Medicine. The study, including the use of tissues and associated clinical data, was approved by the Ethics Committee of Shanghai Municipal Hospital of Traditional Chinese Medicine (No. 2025SHL‐KY‐124‐01).

### 4.14 Tissue Sections and Staining

Human skin melanoma tissue sections for immunohistochemical analysis, (18 melanoma and 18 normal skin tissues) were acquired from Shanghai Zhuoli Biotechnology Co., Ltd. (Shanghai, China). The study including the use of tissue sections and associated clinical data, was approved by the Ethics Committee of Shanghai Zhuoli Biotechtechnology (No. SHLLS‐BA‐22101102). Sections were stained with an anti‐ARHGEF3 antibody for IHC. To minimise bias, section evaluation and data collection were performed in a double‐blinded manner. IHC staining was quantitatively assessed using the H‐score method. Staining intensity was scored as 0 (negative), 1 (weak), 2 (moderate), or 3 (strong). The H‐score was calculated as Σ(pi × i), where pi is the percentage of positive cells at each intensity and i is the corresponding staining score.

For immunofluorescence analysis, 14 human melanoma tissue sections were obtained from the same source and approved under the same ethics protocol. Sections were stained with antibodies against ARHGEF3 (TD4434S, Abmart) and CD8 (Servicebio, China). ImageJ was used for quantitative analysis of the ARHGEF3‐positive area fraction and CD8 density.

### 4.15 Statistical Analysis

All values were presented as the means ± SEM from two to three independent experiments. Schematic illustrations in this study were created using BioRender.com under license. The data were analyzed by using GraphPad Prism version 10. Statistical significance was determined through unpaired Student's t‐test, unequal variance t‐test, Mann–Whitney test, two‐way ANOVA, or log‐rank (Mantel‐Cox) test. Spearman's correlation coefficient was calculated to indicate the correlation between gene expression. A *P*‐value of less than 0.05 was considered significant.

## Author Contributions

Y.L., L.W., Z.Z., and M.S. performed all in vivo and in vitro experiments. X.L. analyzed the clinical datasets. M.S. wrote the manuscript. X.L., Q.B. and M.S. supervised the study.

## Conflicts of Interest

The authors declare no conflicts of interest.

## Ethics Approval and Consent to Participate

All animal experiments were conducted in accordance to the protocol approved by the Ethics Committee of Shanghai University of Traditional Chinese Medicine (License no. SYXK 2020‐0014).

## Supporting information




**Supporting File**: advs75330‐sup‐0001‐SuppMat.pdf.

## Data Availability

All data relevant to the study are included in the article or uploaded as Supplementary Information.
