# Supplementary material for: Tumor‐Intrinsic ARHGEF3 Enhances Antitumor Immunity by Promoting T‐Cell Infiltration and Limiting Myeloid Cell‐Mediated Immunosuppression
Source: Adv Sci (Weinh). 2026 Apr 23;13(38):e23895. doi: 10.1002/advs.202523895 (PMC13335697; doi:10.1002/advs.202523895)
Supplement: Supplementary file 1 — Supporting File: advs75330‐sup‐0001‐SuppMat.pdf. [file ADVS-13-e23895-s001.pdf]

Supplementary Materials for

Tumor-intrinsic ARHGEF3 enhances antitumor immunity by promoting T-cell infiltration and limiting myeloid cell-mediated immunosuppression

Yue Li[1,2#], Lan Wang[4#], Zihao Zhang[5#], Chunmei Qian[1], Ning Li[1], Wei Huang[1], Qian Ba[1*], Xiaojian Liu[3]*, Mayu Sun[1]*

*Corresponding author. Email: mysunsinh@outlook.com (M.S.), liuxj@sjtu.edu.cn (X.L.), qba@shsmu.edu.cn (Q.B.)

This PDF file includes:
Figs. S1 to S13
Table S1

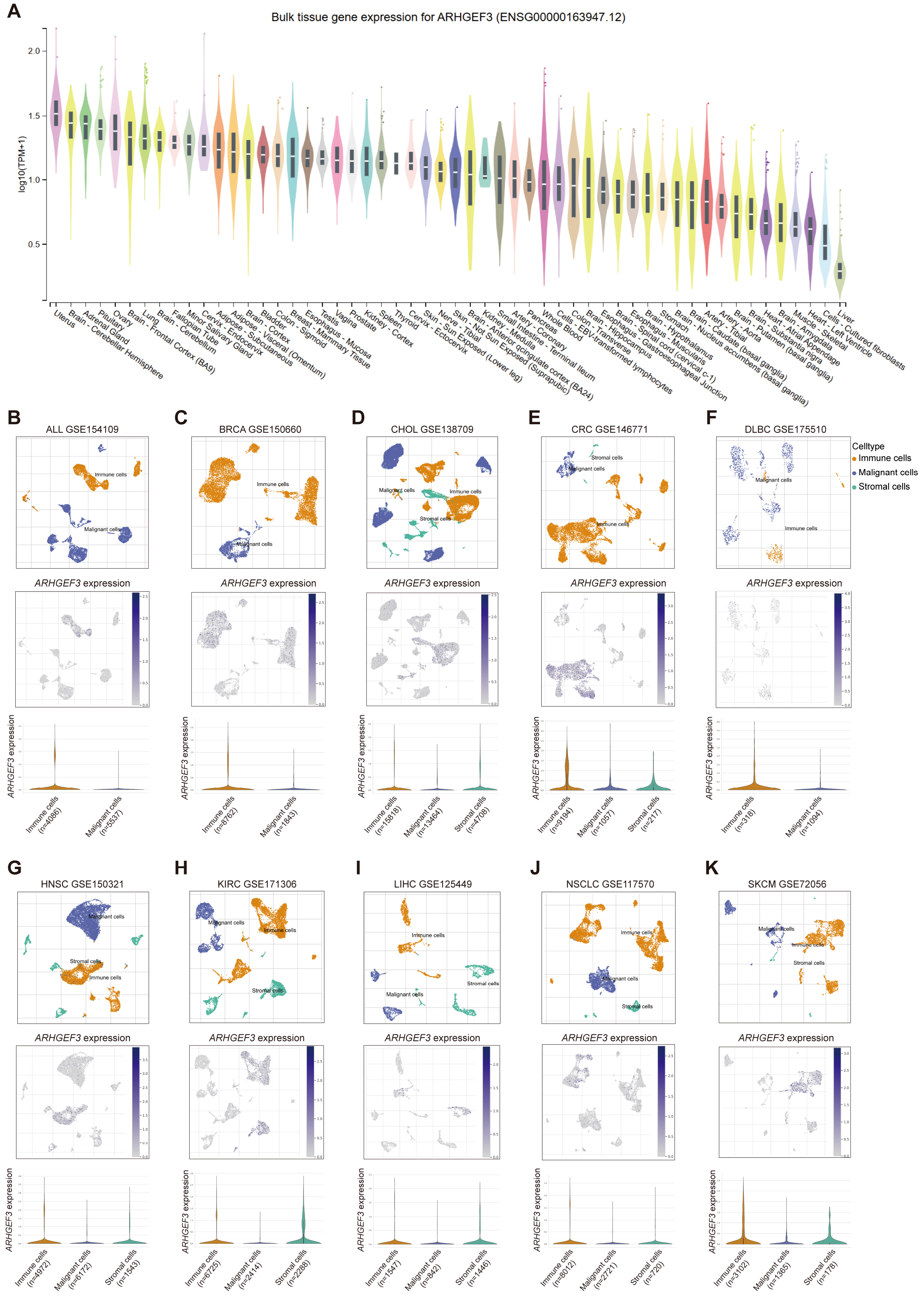

Fig. S1. (A) Violin plots showing *ARHGEF3* expression levels in various human normal tissues. **(B-K)** UMAP plots displaying the clustering of different cell types (Top) and the expression of *ARHGEF3* (middle) in ALL (**B**), BRCA (**C**), CHOL (**D**), CRC (**E**), DLBC (**F**), HNSC (**G**), KIRC (**H**), LIHC (**I**), NSCLC (**J**), and SKCM (**K**). The

violin plot showing ARHGEF3 expression levels (bottom). Data were obtained from the TISCH database. ALL: Acute lymphoblastic leukemia; BRCA: breast invasive carcinoma; CHOL: cholangiocarcinoma; CRC: colorectal cancer; DLBC: diffuse large B-cell lymphoma; HNSC: head and neck squamous cell carcinoma; KIRC: kidney renal clear cell carcinoma; LIHC: liver hepatocellular carcinoma; NSCLC: non–small cell lung cancer; SKCM: skin cutaneous melanoma.

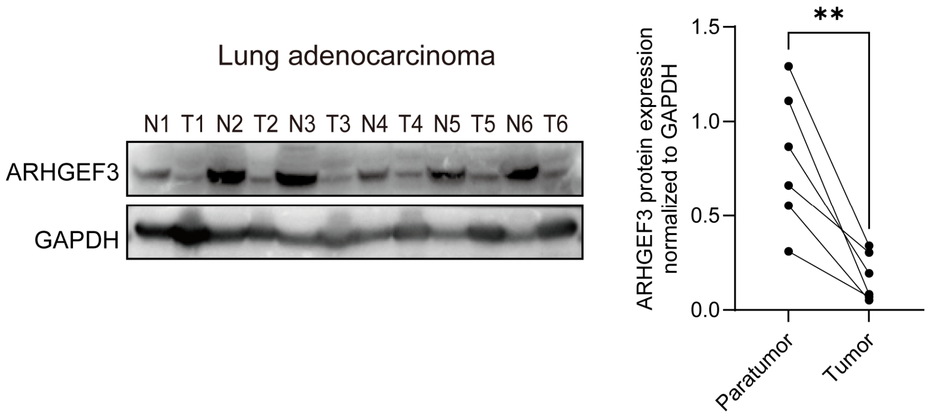

Fig. S2. Western blot analysis of ARHGEF3 expression in paired lung adenocarcinoma and adjacent normal tissues; the right panel shows protein quantification. *P* values were calculated by paired Student's *t*-test.

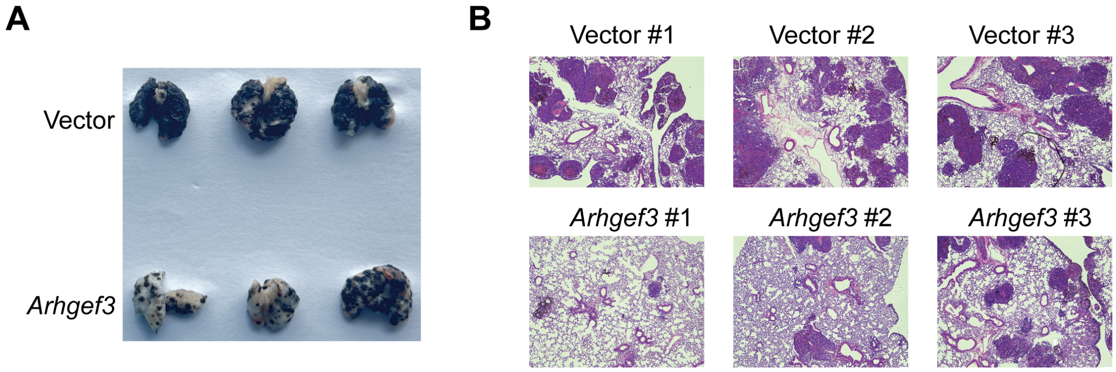

Fig. S3. (**A**) Images of lungs from mice injected via the tail vein with vector control or *Arhgef3*-overexpressing B16F10 cells. Lungs were collected 2 weeks after injection (n = 3 mice per group). (**B**) Representative H&E staining of lung sections from the indicated groups, showing metastatic tumor burden.

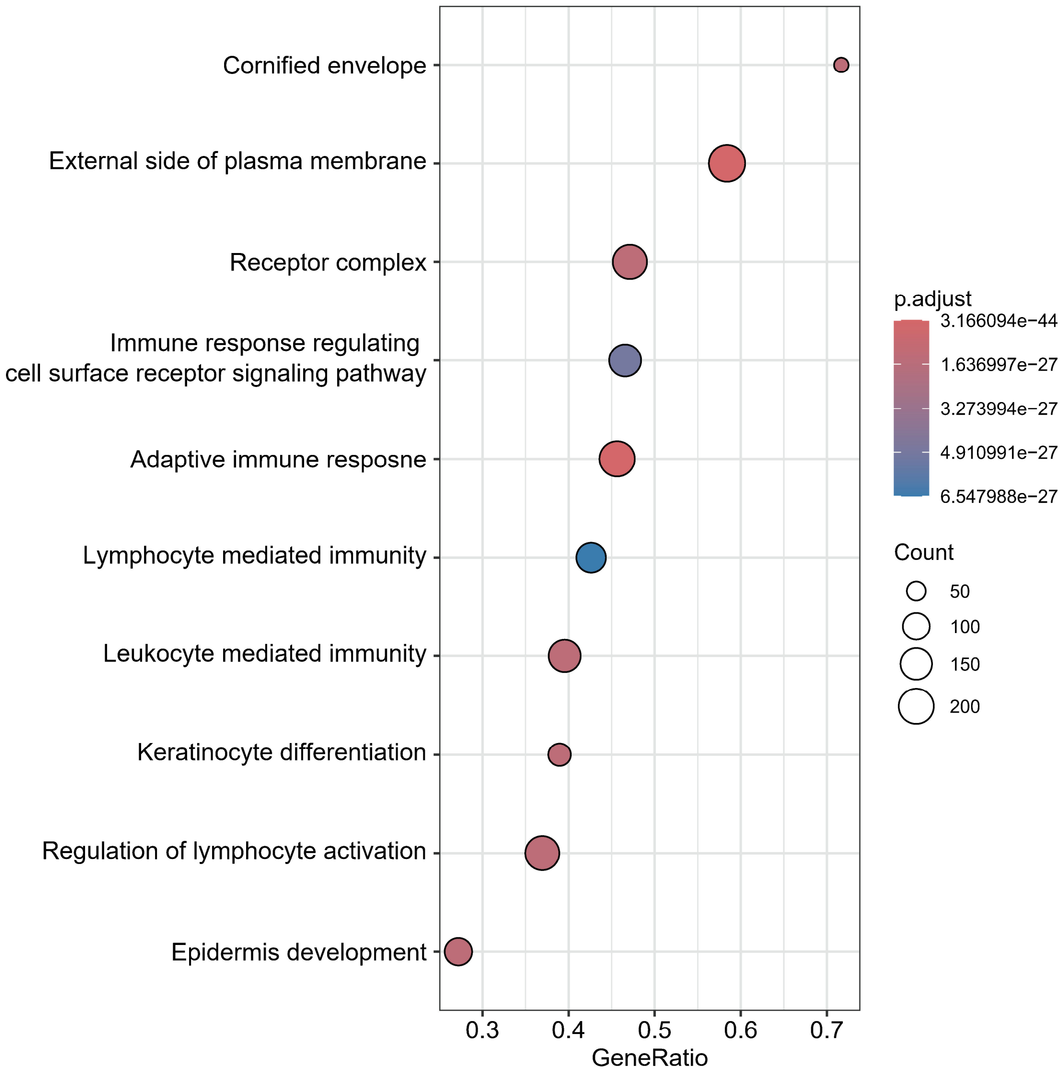

Fig. S4. TCGA-SKCM tumor samples were stratified by *ARHGEF3* expression. Genes were ranked by differential expression (*ARHGEF3*-high vs -low) and tested by GSEA against GO Biological Process gene sets. The top 10 terms were shown.

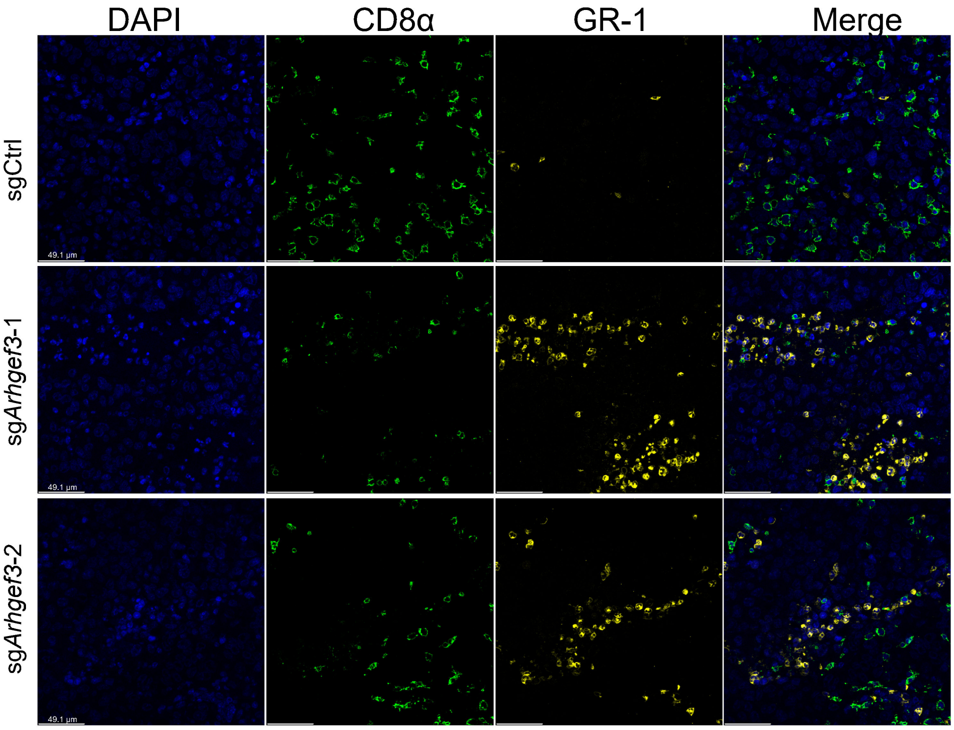

Fig. S5. Representative immunofluorescence images showing intratumoral CD8α and GR-1 expression in *Arhgef3*-sgRNA Hepa1-6 tumor tissues.

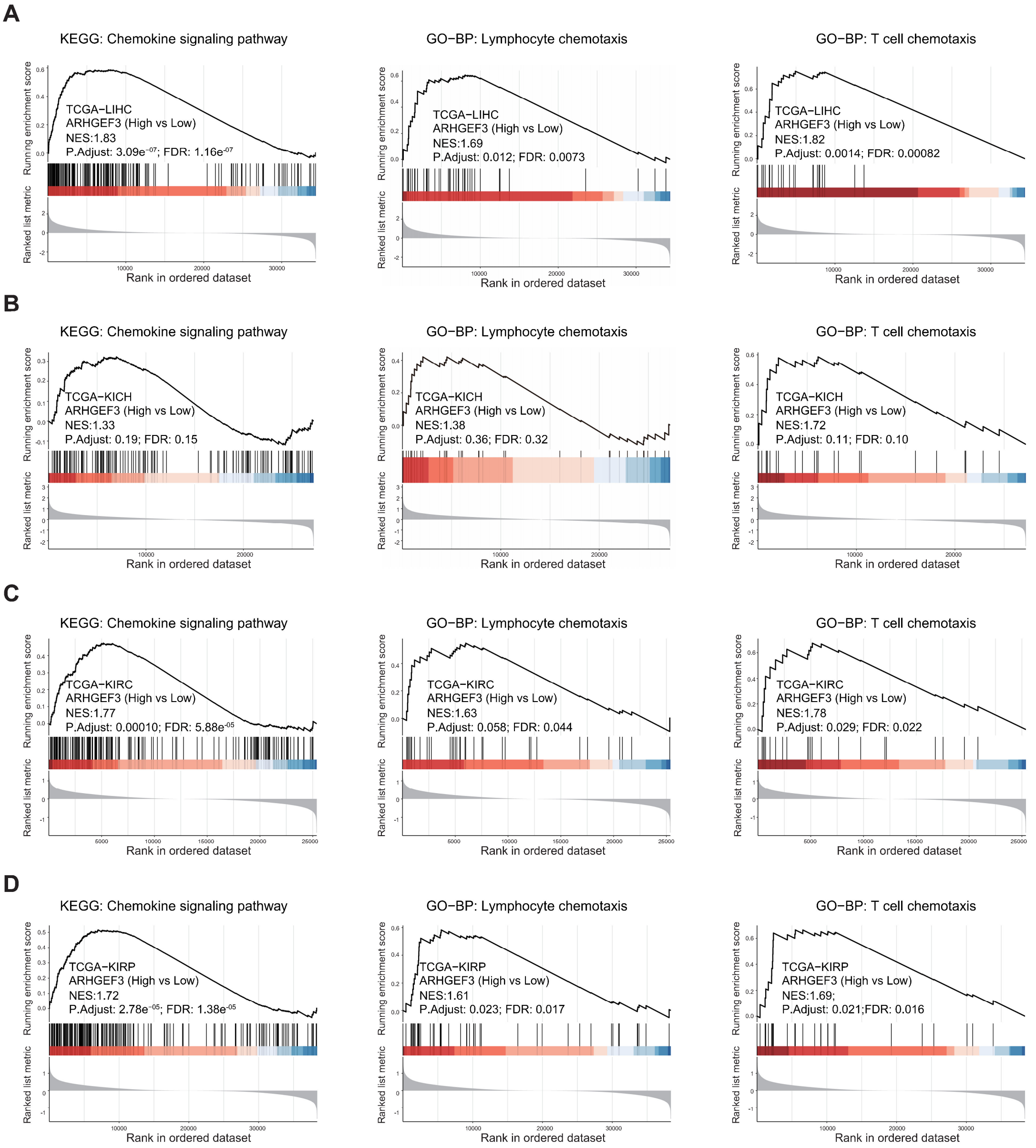

Fig. S6. (**A-D**) GSEA analysis of *ARHGEF3*-high versus *ARHGEF3*-low tumors showing enrichment of lymphocyte chemotaxis, T-cell chemotaxis, and chemokine-mediated signaling across TCGA-LIHC (**A**), -KICH (**B**), -KIRC (**C**), and -KIRP (**D**) cohorts.

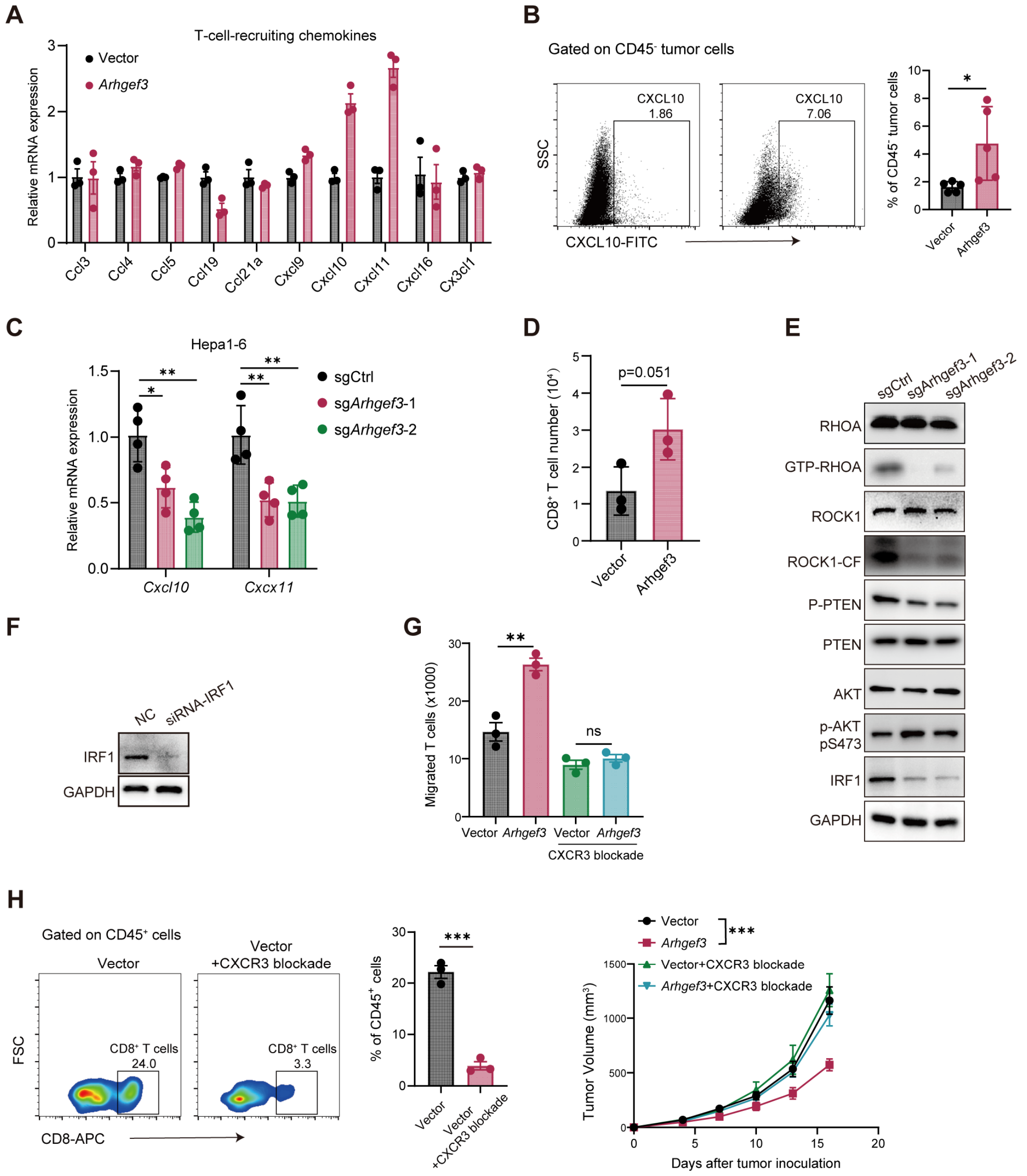

Fig. S7. (**A**) RT-qPCR analysis of T-cell-recruiting chemokines in the indicated B16F10 cells. (**B**) Representative flow cytometry plots (left) and quantification (right) of CXCL10 expression in tumor cells ($CD45^{-}$) from B16F10 tumors. (**C**) RT-qPCR analysis of *Cxcl10*/*Cxcl11* expression in indicated Hepa1-6 cells. (**D**) Quantification of intratumoral T-cell numbers after adoptive transfer of OT-I cells (8×10^5) into T-cell-deficient Rag1$^{-/-}$ mice. (**E**) Protein levels along the RHOA–ROCK1–PTEN–AKT–IRF1 pathway in the indicated Hepa1-6 cells, were determined by western blot. (**F**) The knockdown efficiency of siRNA-*Irf1* in B16F10 cells was determined by western blot.

(**G**) Quantification of migrated T cells recruited by TCM from the indicated B16F10 cells. $CD8^+$ T cells were pretreated with the CXCR3 inhibitor SCH546738 (20 nM) for 1 h before being added to the upper chamber. (**H**) Tumor growth curves of *Arhgef3*-overexpressing B16F10 tumors following CXCR3 blockade. SCH546738 was administered daily by oral gavage at 600 μg per mouse. Representative flow cytometry plots and quantification of tumor-infiltrating T cells after CXCR3 blockade are shown. n=5 mice per group. *P* values were calculated by unpaired Student's *t*-test or two-way ANOVA. (**P*<0.05, ***P*<0.01, ****P*<0.001).

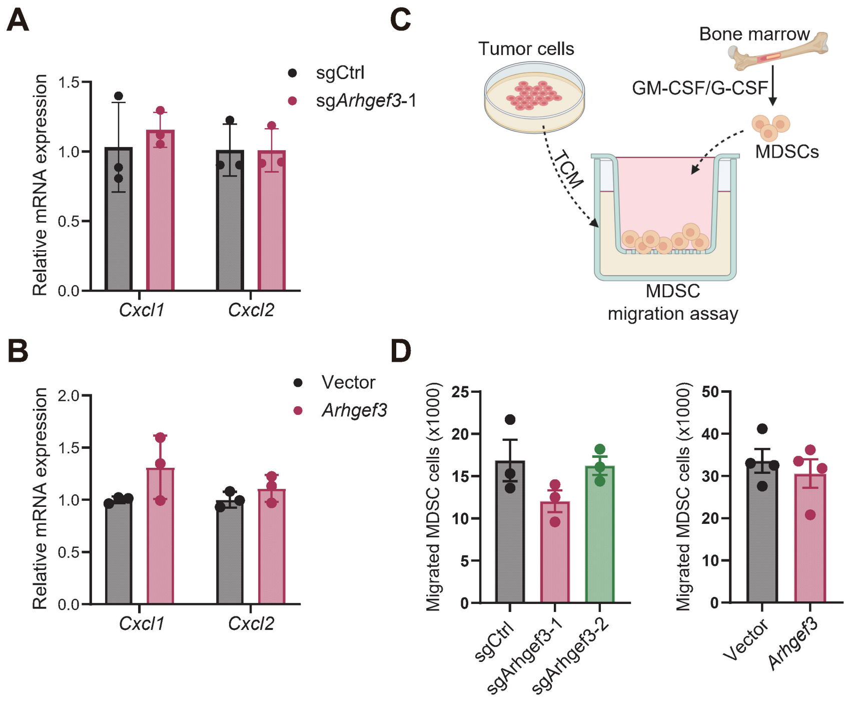

Fig. S8. (**A, B**) RT–qPCR analysis of *Cxcl1* and *Cxcl2* mRNA in *Arhgef3*-sgRNA (**A**) and *Arhgef3*-overexpressing (**B**) B16F10 cells. (**C**) Schematic of the MDSC migration assay; TCM was collected from the indicated B16F10 cells and used as chemoattractant. (**D**) Quantification of migrated MDSCs recruited by TCM from the indicated B16F10 cells.

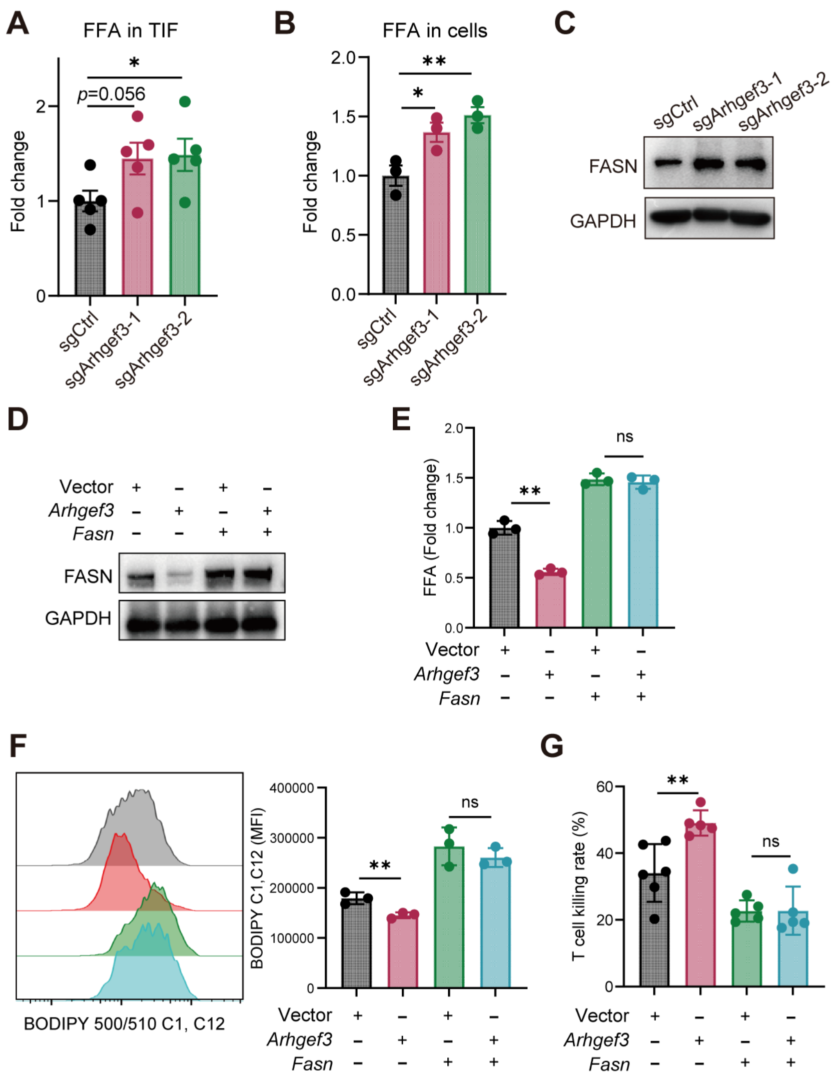

Fig. S9. (**A**) Quantification of free fatty acid (FFA) in tumor interstitial fluid from Hepa1-6 tumors. (**B**) Quantification of free fatty acid in culture supernatants from *Arhgef3*-sgRNA Hepa1-6 cells. (**C**) FASN protein levels in *Arhgef3*-sgRNA Hepa1-6 cells were determined by western blot. (**D-G**) Following *Fasn* overexpression in tumor cells, FASN protein levels (**D**), extracellular free fatty acid levels (**E**), lipid uptake in BM-MDSCs after TCM priming (**F**), and T-cell killing activity (**G**) were assessed. In (**F**), lipid uptake was quantified by the mean fluorescence intensity (MFI) of BODIPY 500/510 C1, C12. *P* values were calculated by unpaired Student's *t*-test. (**P*<0.05, ***P*<0.01).

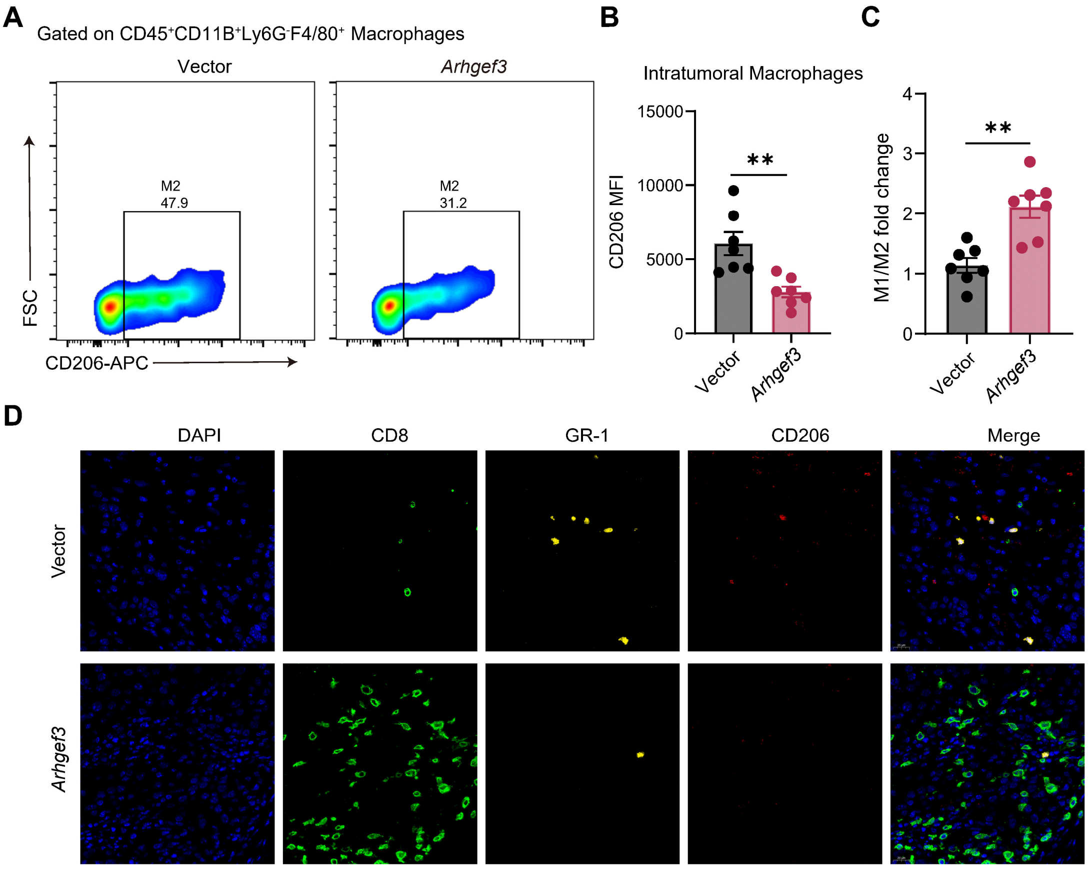

Fig. S10. (**A, B**) Representative flow cytometry plots (**A**) and quantification (**B**) of CD206 expression on intratumoral macrophages in B16F10 tumors. (**C**) Quantification of the intratumoral M1/M2 macrophage ratio. (**D**) Representative immunofluorescence images of *Arhgef3*-overexpressing B16F10 tumor tissues showing CD8α, GR-1, and CD206 expression. *P* values were calculated by unpaired Student's *t*-test. (***P*<0.01).

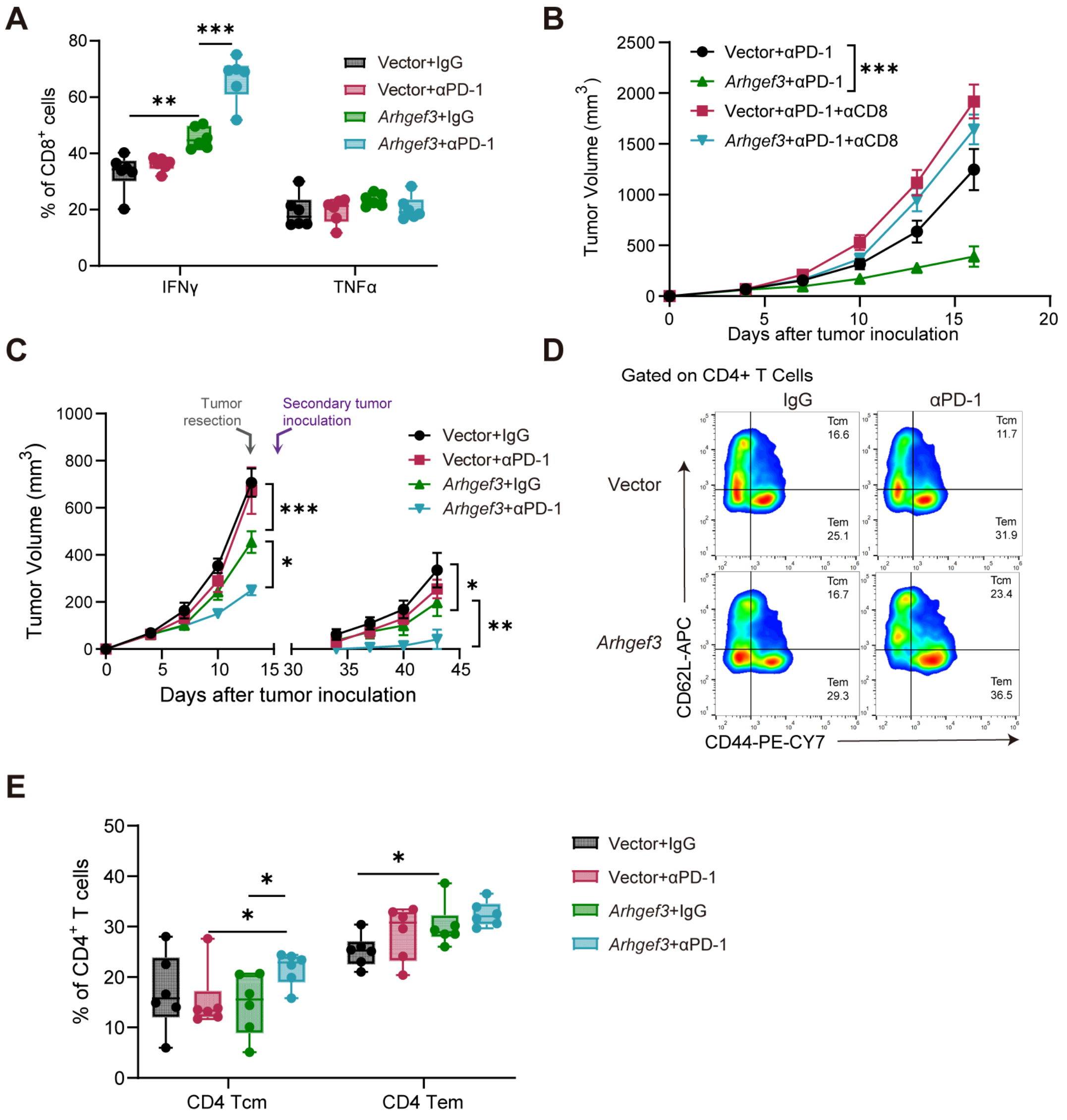

Fig. S11. (**A**) Quantification showing the levels of IFN-γ and TNF-α in intratumoral CD8^{+} T cells. (**B**) Tumor growth curves of *Arhgef3*-overexpressing B16F10 tumors following anti–PD-1 treatment after T-cell depletion with neutralizing antibodies. (**C**) Tumor growth curves in the rechallenge model of *Arhgef3*-overexpressing B16F10 tumors. Mice received anti–PD-1 antibody treatment, and the primary tumors were resected on day 12 after implantation. After a 2-week recovery period, B16F10 cells (4.0×10^{5}) were re-inoculated subcutaneously into the contralateral flank. (**D, E**) Representative flow cytometry plots (**D**) and quantification (**E**) of the frequencies of central memory (Tcm) and effector memory (Tem) subsets within CD4^{+} T cells in B16F10 tumor tissues after anti–PD-1 treatment. *P* values were calculated by unpaired Student's *t*-test. (**P*<0.05, ***P*<0.01, ****P*<0.001).

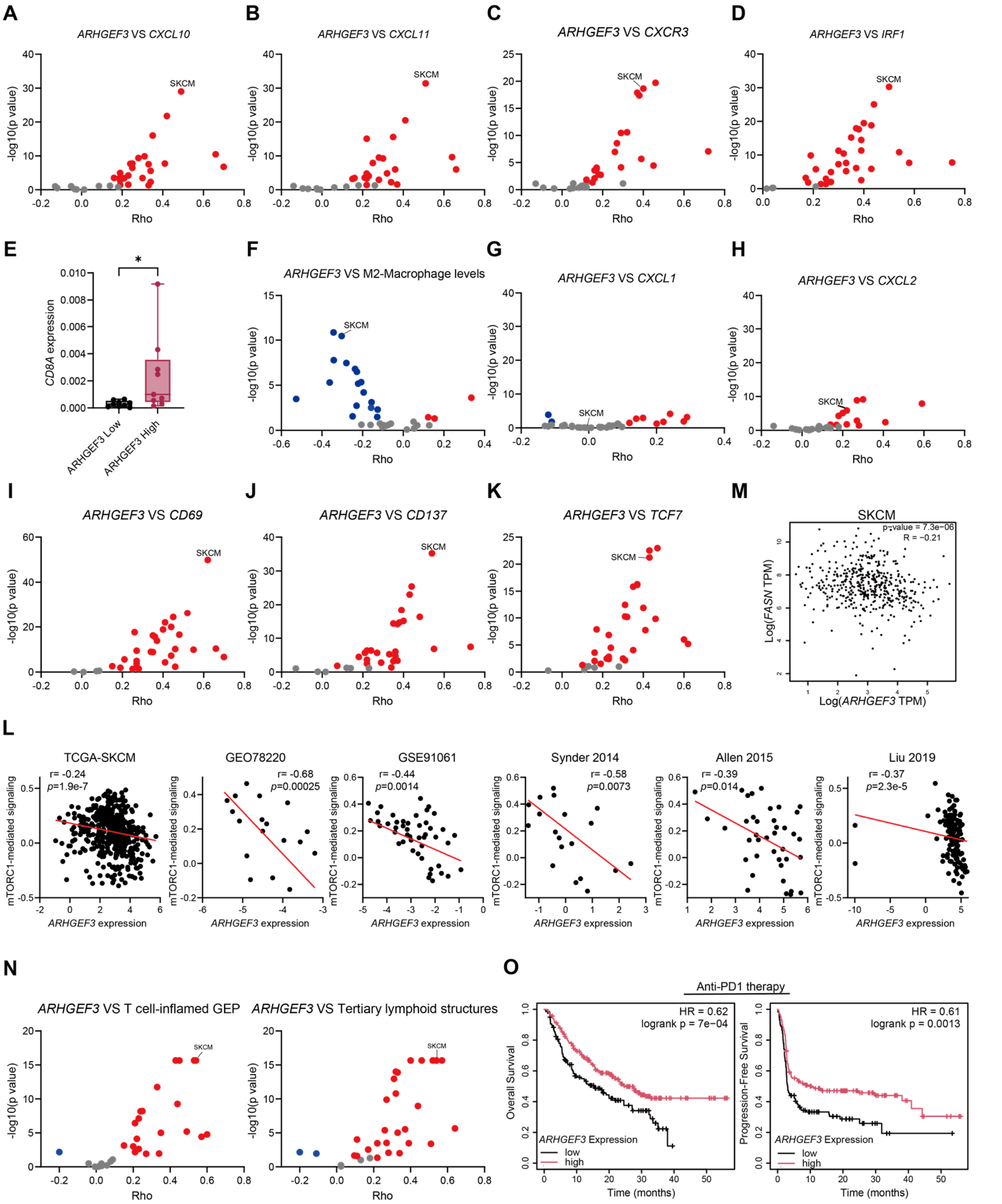

Fig. S12. (**A-D**) Pan-cancer correlations between *ARHGEF3* expression and *CXCL10* (**A**), *CXCL11* (**B**), *CXCR3* (**C**), and *IRF1* (**D**) across TCGA database. (**E**) RT–qPCR analysis of CD8α expression in bladder tumor tissues stratified by *ARHGEF3*. *P* value was calculated by unpaired Student's *t*-test. (**F-K**) Pan-cancer correlations between *ARHGEF3* and M2 macrophage levels (**F**), *CXCL1* (**G**), *CXCL2* (**H**), *CD69* (**I**), *CD137* (**J**), and *TCF7* (**K**) across TCGA database. (**L**) Correlation of ARHGEF3 with mTORC1 signaling in TCGA-SKCM and multiple melanoma cohorts. (**M**) Correlation analysis of ARHGEF3 expression with FASN in TCGA-SKCM database. (**N**) Pan-

cancer correlations of *ARHGEF3* with the T cell–inflamed GEP and tertiary lymphoid structures (TLS) across TCGA database. (**O**) Overall survival (OS) and progression-free survival (PFS) in anti–PD-1–treated cohorts stratified by *ARHGEF3* expression. *P* values were calculated by Spearman's rank correlation for correlation analyses, and the log-rank (Mantel-Cox) test for survival curves. (**P*<0.05).

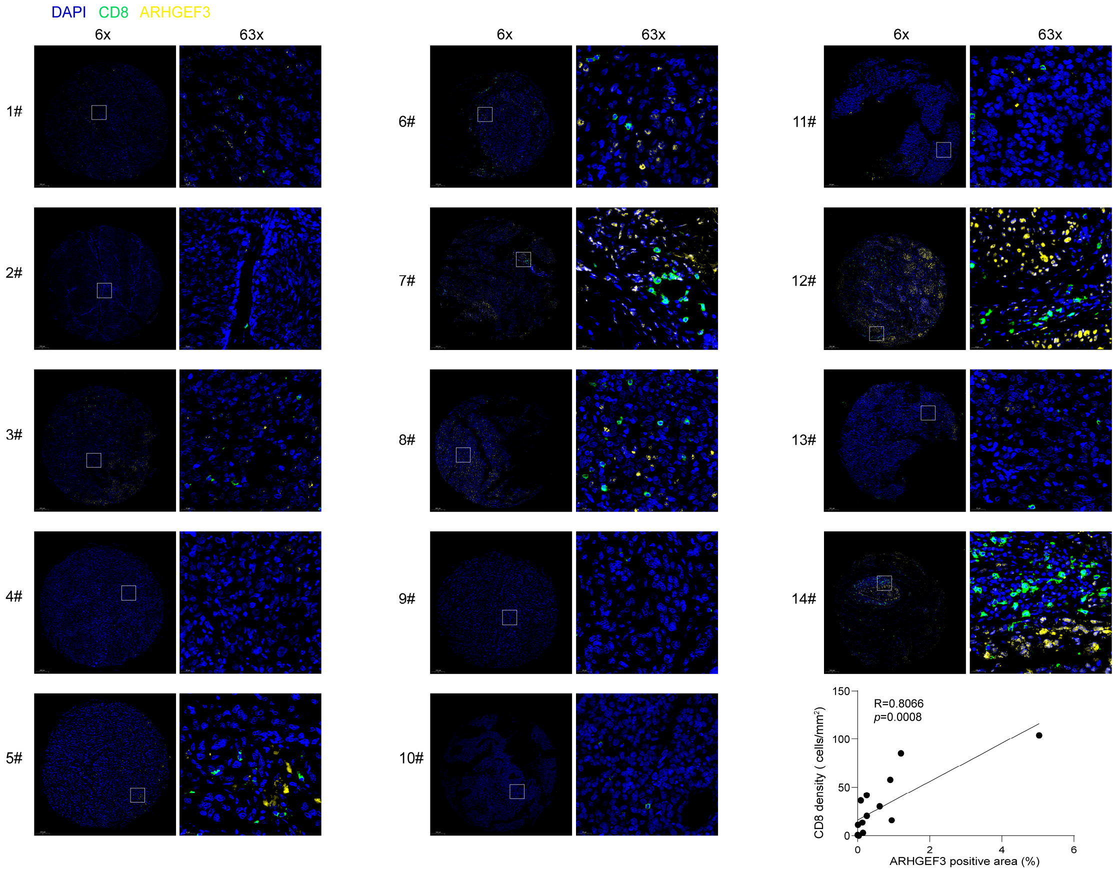

Fig. S13. Immunofluorescence staining of ARHGEF3 and CD8 in human melanoma tissues (n = 14), shown at 6× and 63× magnification. Nuclei were counterstained with DAPI (blue). ARHGEF3 is shown in yellow and CD8 in green. The ARHGEF3-positive area fraction and CD8 density were quantified, and their association was evaluated by Spearman's correlation analysis.

Supplementary table 1. Primers for qRT-PCR

Species	Gene	Forward sequence	Reverse sequence
Mouse	*Cd69*	CGGAAAATAGCTCTTCACATCTG	GAGGACCACTATTAACACAGCC
	Gzmb	CCACTCTCGACCCTACATGG	GGCCCCCAAAGTGACATTTATT
	Ifng	ATGAACGCTACACACTGCATC	CCATCCTTTTGCCAGTTCCTC
	Pdcd1	ACCCTGGTCATTCACTTGGG	CATTTGCTCCCTCTGACACTG
	Tox	GCTTGATGTGAGAGTGAAATGG	GCTCATATACATGTTCTCCCCG
	Tcf7	AGCTTTCTCCACTCTACGAAC	AATCCAGAGAGATCGGGGGTC
	Tgfb	ATGTCACGGTTAGGGGCTC	GGCTTGCATACTGTGCTGTATAG
	Cd274	GCTCCAAAGGACTTGTACGTG	TGATCTGAAGGGCAGCATTTC
	Il-10	TGCACTACCAAAGCCACAAGGCAG	AGTAAGAGCAGGCAGCATAGCAGT
	Arg-1	ACCTGGCCTTTGTTGATGTCCCTA	AGAGATGCTTCCAACTGCCAGACT
	Vegfa	GCACATAGAGAGAATGAGCTTCC	CTCCGCTCTGAACAAGGCT
	Cxcl10	TCAGCACCATGAACCCAAG	CTATGGCCCTCATTCTCACTG
	Cxcl11	ATGGCAGAGATCGAGAAAGC	TGCATTATGAGGCGAGCTTG
	Cxcl1	AACCGAAGTCATAGCCACAC	CAGACGGTGCCATCAGAG
	Cxcl2	AATGCCTGAAGACCCTGC	TTTTGACCGCCCTTGAGAG
	Fasn	CCCCTCTGTTAATTGGCTCC	TTGTGGAAGTGCAGGTTAGG
	Cxcl9	AGTCCGCTGTTCTTTTCCTC	TGAGGTCTTTGAGGGATTTGTAG
	Ccl5	GGGTACCATGAAGATCTCTGC	TCTAGGGAGAGGTAGGCAAAG
	Ccl3	GATTCCACGCCAATTCATCG	TTCAGTTCCAGGTCAGTGATG
	Ccl4	AAACCTAACCCCGAGCAAC	CGGGAGGTGTAAGAGAAACAG
	Ccl19	CGCATCATCCGAAGACTGAAG	TTTACTCAAGACACAGGGCTC
	Ccl21a	GGGAACCTCTAAGTCTGGAAAG	TTGAGGGCTGTGTCTGTTC
	Cx3cl1	TCTTCCATTTGTGTACTCTGCT	GGACTCCTGGTTTAGCTGATAG
	Cxcl16	GTTGCAGTCCAAAAGCGTG	GTCTGGGTACTGGCTTGAG
	Actb	AGAGGGAAATCGTGCGTGAC	CAATAGTGATGACCTGGCCGT
Human	*ARHGEF3*	GCCTGCCATACTGAGTCTATG	CTTTAGGAGAGGGAAACATGGG
	CD8A	ATGGCCTTACCAGTGACCG	AGGTTCCAGGTCCGATCCAG
	β-ACTIN	CTCGCCTTTGCCGATCC	TCTCCATGTCGTCCCAGTTG
